# Cross-linking breast tumor transcriptomic states and tissue histology

**DOI:** 10.1016/j.xcrm.2023.101313

**Published:** 2023-12-19

**Authors:** Muhammad Dawood, Mark Eastwood, Mostafa Jahanifar, Lawrence Young, Asa Ben-Hur, Kim Branson, Louise Jones, Nasir Rajpoot, Fayyaz ul Amir Afsar Minhas

**Affiliations:** 1 Tissue Image Analytics Centre, University of Warwick, Coventry, UK; 2 Warwick Medical School, University of Warwick, Coventry, UK; 3 Cancer Research Centre, University of Warwick, Coventry, UK; 4 Department of Computer Science, Colorado State University, Fort Collins, CO, USA; 5 Artificial Intelligence & Machine Learning, GlaxoSmithKline, San Francisco, CA, USA; 6 Barts Cancer Institute, Queen Mary University of London, London, UK; 7 The Alan Turing Institute, London, UK

**Keywords:** computational pathology, genotype to phenotype mapping, spatial transcriptomics, graph neural networks, topic modelling, gene groups, transcriptomics, breast cancer, receptor status prediction

## Abstract

Identification of the gene expression state of a cancer patient from routine pathology imaging and characterization of its phenotypic effects have significant clinical and therapeutic implications. However, prediction of expression of individual genes from whole slide images (WSIs) is challenging due to co-dependent or correlated expression of multiple genes. Here, we use a purely data-driven approach to first identify groups of genes with co-dependent expression and then predict their status from WSIs using a bespoke graph neural network. These gene groups allow us to capture the gene expression state of a patient with a small number of binary variables that are biologically meaningful and carry histopathological insights for clinical and therapeutic use cases. Prediction of gene expression state based on these gene groups allows associating histological phenotypes (cellular composition, mitotic counts, grading, etc.) with underlying gene expression patterns and opens avenues for gaining biological insights from routine pathology imaging directly.

## Highlights

• Data-driven discovery of co-expressing gene groups in breast cancer
• Histological imaging-based prediction of gene groups via deep learning
• Identification of phenotypic correlates of gene expression in histological imaging
• Clinical and therapeutic impact of gene groups and their visual patterns identified

## Graphical abstract

**Figure:**
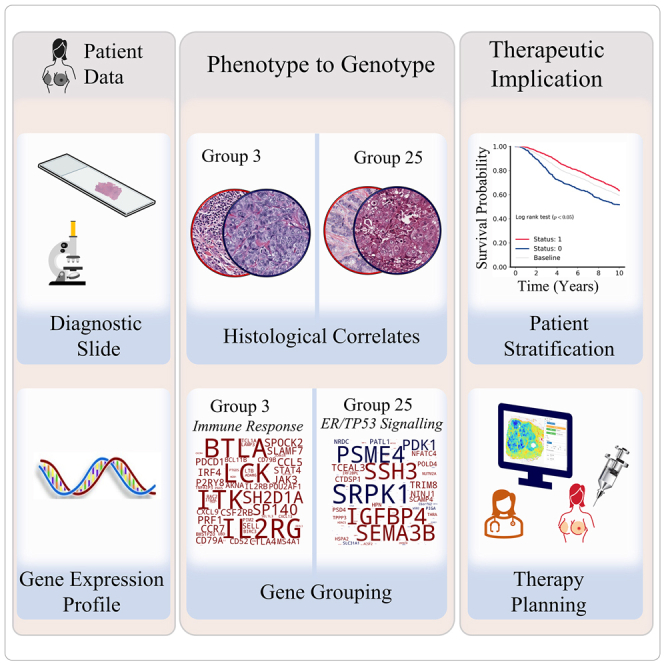

## Teaser

Dawood et al. uncover associations in the expression of genes in breast tumors and present a deep learning approach for prediction of transcriptomic states from routine histology images, enabling an improved understanding of genotype to phenotype mapping and clinicopharmacological parameters in breast cancer.

## Introduction

Cancer is a clonal disease in which genetic alterations directly or indirectly alter gene expression, biological pathways, and proteins activity leading to phenotypic changes in the spatial organization of the tumor microenvironment.^1^ Consequently, associating histological and molecular patterns is crucial for understanding disease mechanisms and clinical decision-making.^2^ During histopathology examination, a tumor section stained with hematoxylin and eosin (H&E) is visually examined for features such as mitotic counts, nuclear pleomorphism, epithelial tubule formation, necrosis, and tumor-infiltrating lymphocytes to develop a spatially informed histological profile of the disease. Similarly, gene expression analysis based on molecular tests such as PAM50,^3^^,^^4^ Oncotype-Dx,^5^ and Mammaprint^6^ can also be used for patient subtyping. Gene expression profiling based on such limited gene assays or from bulk RNA sequencing (RNA-seq)^7^ and single-cell RNA-seq^8^^,^^9^ plays a key role in understanding the genetic basis of cancer and discovery of new therapeutic targets. However, such technologies are unable to capture spatial heterogeneity in the expression profile of genes across a tumor section. Spatial profiling of a tumor transcriptome is typically achieved using spatially resolved transcriptomics technologies.^10^ However, such technologies are generally costly and offer low resolution in terms of spatial details or genes.^11^^,^^12^ Consequently, there is a need for cross-linking gene expression and spatial histological imaging profiles to gain a more in-depth understanding of latent factors associated with the disease.

In an attempt to achieve this goal, recent advancements in deep learning for computational pathology have demonstrated that prediction of expression profiles of genes is possible from whole slide images (WSIs) of H&E-stained tissue sections.^13^^,^^14^^,^^15^ For example, Schmauch et al.^15^ proposed a deep learning method called HE2RNA for predicting gene expression profiles from WSIs. Similarly, Wang et al.^16^ proposed a deep learning method for predicting the expression profile of 17,695 genes from WSIs. For each of the 17,695 genes, the authors have tiled the WSIs into patches and then trained and optimized an Inception V3 for predicting tile-level and WSI-level expression. Most recently, an attention-based method called tRNAsformer has been proposed for predicting the expression level of individual gene from WSIs in kidney cancer.^17^

The vast majority of image-based RNA-seq expression prediction methods focus on associating tissue morphology with the expression level of individual genes.^15^^,^^16^^,^^17^ This is typically done by designing a machine learning pipeline in which the input is a WSI and the output is the expression level of a single gene. However, due to the nature of the biological mechanisms underlying gene expression, genes usually show co-dependent or correlated expression. Consequently, it is, in general, not possible to associate the predicted expression of a single gene from the input WSI to that gene alone. Furthermore, an observed phenotypic effect cannot solely be pinpointed to the known function of a single gene as, typically, it will be a collective effect exhibited by the expression of functionally inter-related genes and a single gene may be associated with multiple functions.^18^ Therefore, instead of predicting the phenotypic effect of a single gene from WSIs, it is more meaningful to predict the expression of groups of genes that act concomitantly and exhibit coherent patterns of expression across samples.

In contrast with existing research in this domain that focuses on prediction of expression level of individual genes from WSIs, in this work we first characterize the gene expression state of a patient in terms of a small number of binary latent factors or gene groups that are discovered in a purely data-driven manner. These can be viewed as overlapping groups of related genes whose expression shows significant inter-dependence across samples. The motivation behind such gene grouping is that, though co-expression is not causation, genes with co-dependent expression show coordinated responses across a significant subgroup of patients, hinting that these genes may be part of an underlying biological pathway, protein complex, or disease subtype.^19^ We have shown that the discovered gene groups are clinically and pathologically relevant in terms of their association with survival, breast cancer receptor status, histopathological phenotypes, cancer driver genes mutations, biological pathways enrichment and underlying protein-protein interactions, and therapeutic decision-making. We then propose a bespoke multi-output graph neural network-based computational pathology pipeline to predict the expression state of a patient in terms of these latent factors from their WSIs. This enables the identification of spatial histological patterns associated with individual latent factors, as well as the overall gene expression profile of a patient. Finally, we have shown that image-based predicted gene group statuses can be used as a latent representation for the prediction of several other downstream clinical tasks, such as patient subtyping and driver gene and pathway alteration status.

## Results

### Analytic workflow

**Figure 1:**
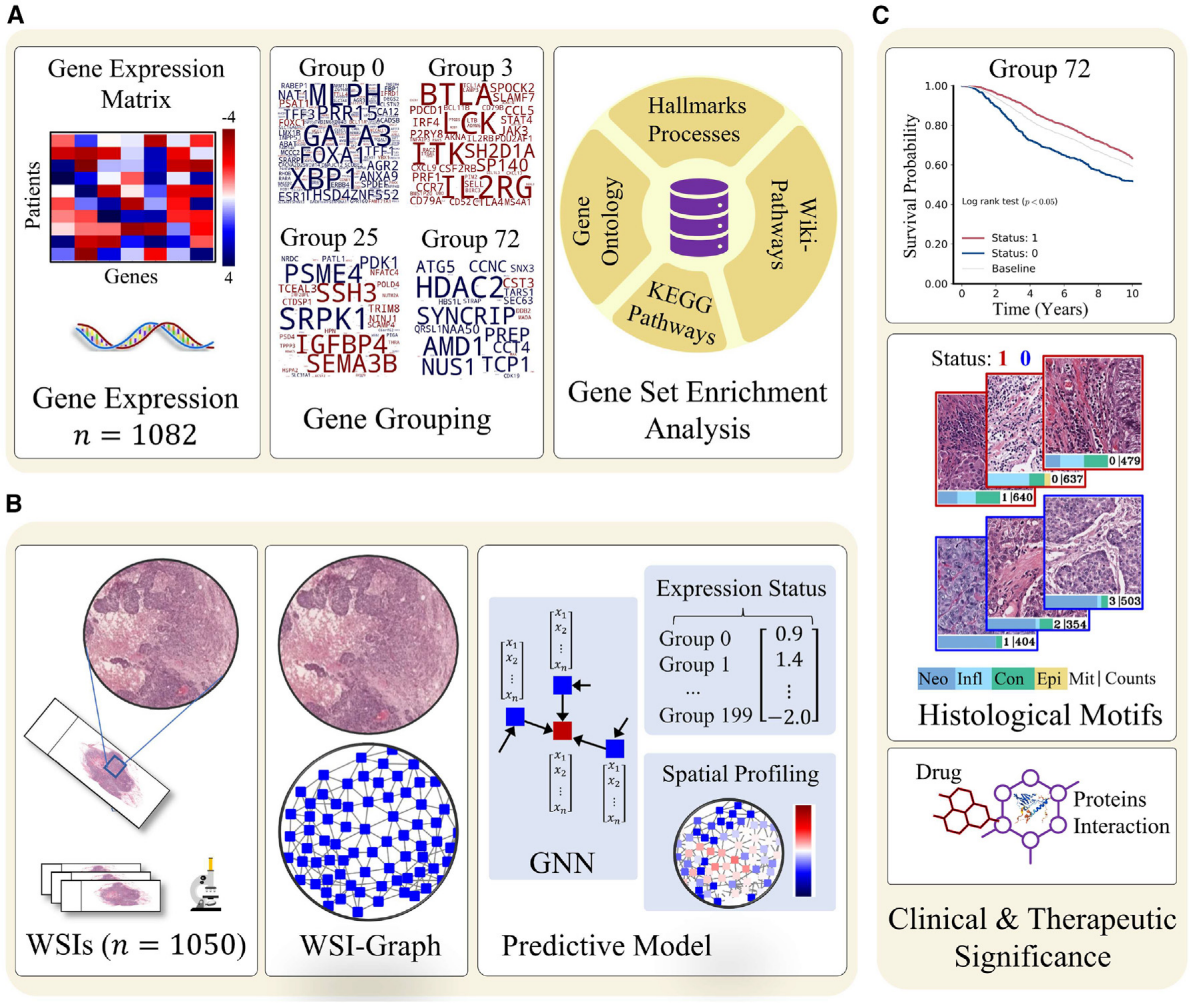
Analytic workflow for patient gene expression state prediction from WSIs (A) Workflow of data-driven discovery of gene groups and their pathological significance is shown. We first identified 200 binary latent factor or gene groups from the gene expression data in a data-driven manner. A gene group can be viewed as overlapping group of genes that exhibit coherent patterns of expression across sample. Word clouds demonstrate the gene composition of different gene groups. The color of the gene indicates whether its median expression across patients is high (red) or low (blue) when gene group status = 1. Afterward, we assessed the biological significance of the genes grouped in different gene groups through GSEA. (B) The proposed ${SlideGraph}^{\infty }$ pipeline for prediction of gene groups status from WSIs. We first construct graph representation of a WSI and then feed it into a graph neural network (GNN) for predicting WSI-level and spatially resolved expression status of these 200 gene groups. (C) Identification of clinically relevant gene groups in terms of association with survival and their associated histological motifs. Histology image-based inference of personalized medication by analyzing PPIs and PDIs of gene groups.

As shown in Figure 1, we performed gene expression analysis of the TCGA breast cancer (TCGA-BRCA) cohort (n = 1082) to identify 200 groups of genes such that the expression of genes in the same group is maximally co-dependent. This allows us to capture the inter-dependence between expression profiles of different genes and represent the gene expression state of a given patient in the form of 200 binary variables each corresponding with a single group. To underscore the clinical, therapeutic, and biological significance of each gene group, we computed the association of patient gene group status with survival, enrichment for biological pathways and cancer hallmark processes, and protein-protein and drug-protein interactions.

We then used our bespoke graph neural network-based pipeline that takes a WSI as input and predicts the binary status of 200 gene groups simultaneously in an end-to-end manner. This allows us to model the complete gene expression profile of a patient and identify histological imaging patterns associated with each gene group. Furthermore, the proposed approach allows spatially resolved cross-linking of discovered gene groups with visual information contained in the WSI. The interactive visualization portal for the proposed approach (called Histology Gene Groups Xplorer [HiGGsXplore]) is available at: (http://tiademos.dcs.warwick.ac.uk/bokeh_app?demo=HiGGsXplore). Finally, we assessed the generalization performance of the proposed approach on three independent validation cohorts (METABRIC, CPTAC-BRCA, and ABCTB) and compared our prediction results with other existing pipelines.

### Data-driven discovery of gene groups based on co-dependent expression

To capture multivariate nonlinear relationships in gene expression patterns across patient samples, we employed Correlation Explanation (CorEx) on RNA-seq data of the TCGA-BRCA cohort. CorEx can be used to model the underlying dependency structure of a dataset by identifying groups of random variables that in the context of this application can intuitively be viewed as a manifestation of underlying covarying patterns of gene expression profiles of different genes across patients. The input to CorEx is a 1,082×5,676 matrix where each row is the normalized gene expression score of 5,676 genes with high expression variance or mutation frequency for each of the 1,082 patients. For these data, CorEx identified 200 gene groups that can explain the co-dependence between gene expression patterns observed in the data with minimal loss of information. This allows us to represent the gene expression state of each patient in terms of these 200 binary variables, rather than the expression of all individual genes. As these binary gene expression group statuses are identified in a purely empirical manner directly from gene expression data, the expected impact of any human observation biases on the definition of these gene groups is minimal. Furthermore, a single gene can be associated with multiple gene groups, which is desirable from a biological point of view, as gene products often perform multiple roles within a cell and can be part of multiple interaction networks.^20^

**Figure 2:**
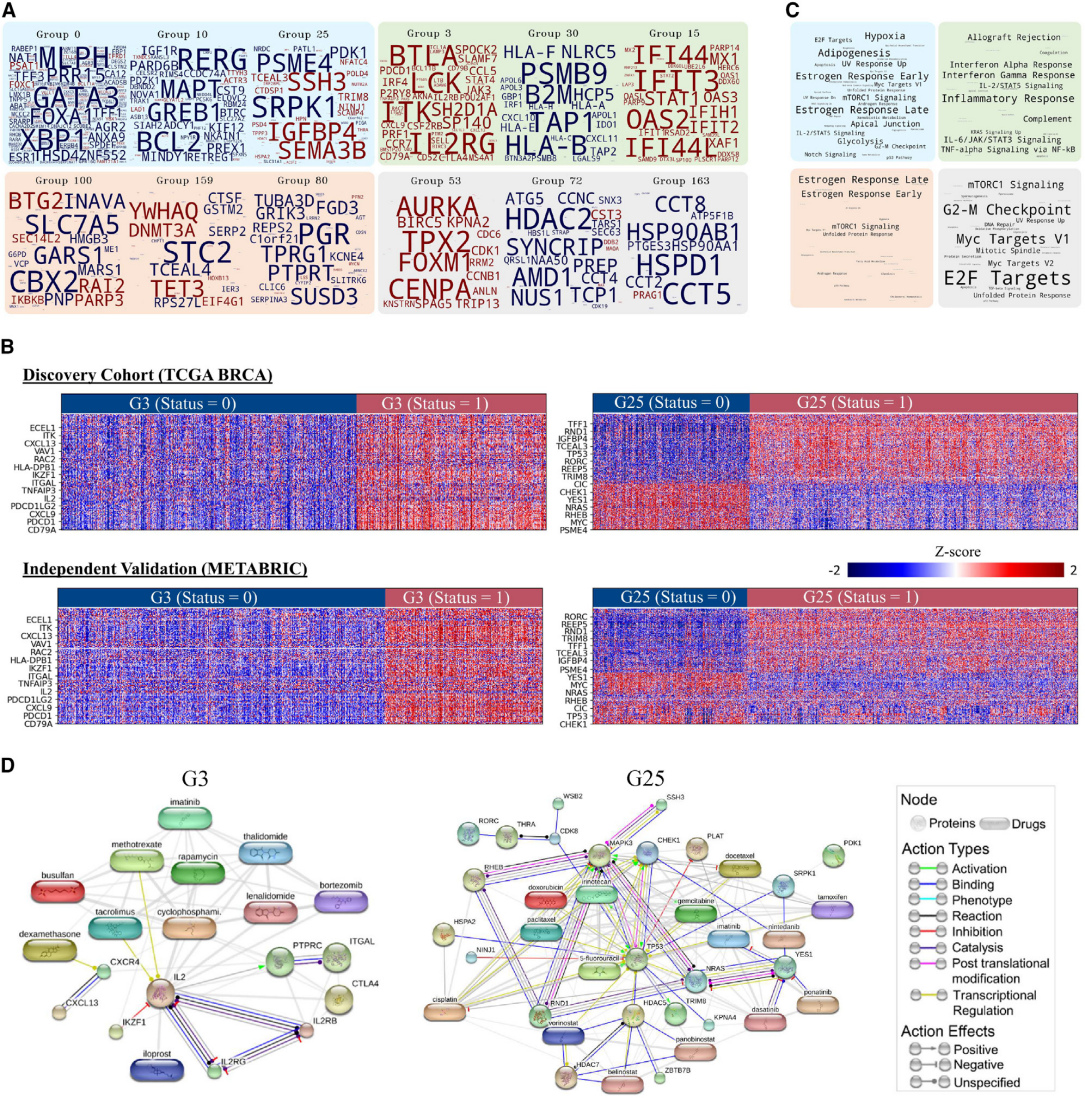
Data-driven discovery of gene groups, their biological and therapeutic significance (A) Word clouds demonstrating the gene composition of different gene groups. The color of the gene indicates whether its median expression across patients is high (red) or low (blue) when gene group status = 1. The font size of a gene within a group is proportional to the amount of information that the gene status provides about a particular gene. (B) Gene expression profile and group status of genes (one per row) for all patients (one per column) in G3 and G25 are shown. (C) Enriched terms for hallmark processes in similar gene groups (note color in A) are shown, with font sizes proportional to the number of gene groups that show enrichment for a certain process. (D) PPI and PDI of selected genes in G3 (left plot) and G25 (right plot) are shown. Nodes shown in circles represent proteins, while the rounded rectangle shapes represent drugs. The edges between nodes show different types of interaction and potential therapeutic targeting.

The gene composition of a selected number of gene groups is shown as word clouds in Figure 2A. For example, the binary status of gene group (G0) is defined primarily based on the expression patterns of a set of genes (*MLPH*, *GATA3*, *XBP1*, *FOXA1*, *TFF3*, *ESR1*, etc.). The exhaustive list of genes grouped in all 200 groups is provided in the Supplementary data. Figure 2B illustrates the underlying co-dependent expression of genes grouped in a selected gene group along with their group status. The heatmaps show that the expression level of genes in G3 and G25 are significantly co-dependent across patients. For instance, for patients with G3 = 1, the expression level of *ITK*, *IL2*, *PDCD1* or *PD1*, *ITGAL*, *PDCD1LG2* or *PD-L2*, and several other genes are high, whereas, for patients with G3 = 0, these genes show under-expression, as shown in Figure 2. For G25, a consistent trend in gene expression can be seen between status = 0 and 1 patients, with more extreme expression (high or low) for patients with G25 = 0 in comparison with patients with G25 = 1. We validated the CorEx model trained on TCGA-BRCA cohort on two independent cohorts (METABRIC and CPTAC-BRCA) and found a consistent association between gene groups status and the expression pattern of set of genes in that group in the independent cohorts as well (see Figures 2B and S1). Additionally, to eliminate the possibility of potential batch effects we ensured that the gene groups obtained from CorEx do not show high degree of predictability of tissue source site (see Table S1).

This key result lends support to the motivation of this work, i.e., the expression level of multiple genes is significantly and consistently inter-dependent and the overall gene expression state of a patient can be characterized by a small number of latent factors. It also highlights the fact that it is not possible to disentangle the expression status of individual genes and consequently associate an observed phenotype, say in a WSI, with the status of a single gene. We next investigated the pathological significance of these gene groups and analyze their predictability from WSIs.

### Pathological significance of gene groups

Here, we discuss the clinicopathological significance of gene groups to understand the implications of these latent factors for clinical decision-making before analyzing their predictability from imaging.

#### Association of gene groups with cancer hallmarks and biological pathways

Through gene set enrichment analysis (GSEA), we found genes from several gene groups associated with known cancer hallmark processes and biological pathways. In Figure 2C, we show the enriched terms for cancer hallmark processes in selected gene groups. For example, genes in G0, G10, and G25 show enrichment for Estrogen early and late response, KRAS and mTORC1 signaling, unfolded protein response, p53 pathway, and several other hallmark processes. Similarly, we found genes from G3, G15, and G30 associated with an inflammatory response, interferon alpha and gamma response, and several other cancer hallmark processes. Apart from cancer hallmark processes, we found genes from a number of gene groups enriched for several biological processes (e.g., T cell receptor signaling for G16 and G20, mitogen-activated protein kinase cascade for G8 and G148, negative regulation of programmed cell death [PD] for G2 and G19, etc.) and Kyoto Encyclopedia of Genes and Genomes (KEGG) pathways (e.g., *PD-L1* expression and *PD-1* checkpoint pathway for G3, G16, and G71, JAK-STAT, and PI3K-Akt signaling pathway for G7 and G72, Th2 and Th17 cell differentiation for G16, G20, and G27, etc.). A comprehensive list of the associations between all gene groups and cancer hallmark processes, biological processes, and KEGG pathways is provided in the Supplementary data.

#### Gene groups capture clinically important protein-protein and protein-drug interactions

We analyzed the protein-protein interaction (PPI) and protein-drug interaction (PDI) of genes in several gene groups with the end goal of identifying which groups involve proteins that show interaction with known anticancer agents. Figure 2D shows the PPI and PDI of a selected number of genes from G3 and G25. Regarding G3, interaction between *IL2*, *IL2RB*, and *IL2RG* can be seen (Figure 2D, left), which is expected as *IL2* regulates immunity by teaming up with *IL2RB* and *IL2RG*.^21^^,^^22^ Similarly, interaction of tacrolimus, an immunosuppressive and anti-inflammatory macrolide that targets the CD4^+^ cells can be seen with *IL2*. In reference to G25 (Figure 2D, right), TRIM8 a member of the tripartite motif-containing (TRIM) binding with TP53 can be seen, which has been shown to play a role in regulating TP53/p53-mediated pathway.^23^ Similarly, interaction of *YES1*, a targetable oncogene can be seen with drugs such as dasatinib, ponatinib, nintedanib, and imatinib. Since our analysis indicates significant alignment between gene groups status and the expression level of proteins (see Figure S2), gene group status can provide some insights into these interactions.

#### Patient stratification into high and low risk using gene groups status

**Figure 3:**
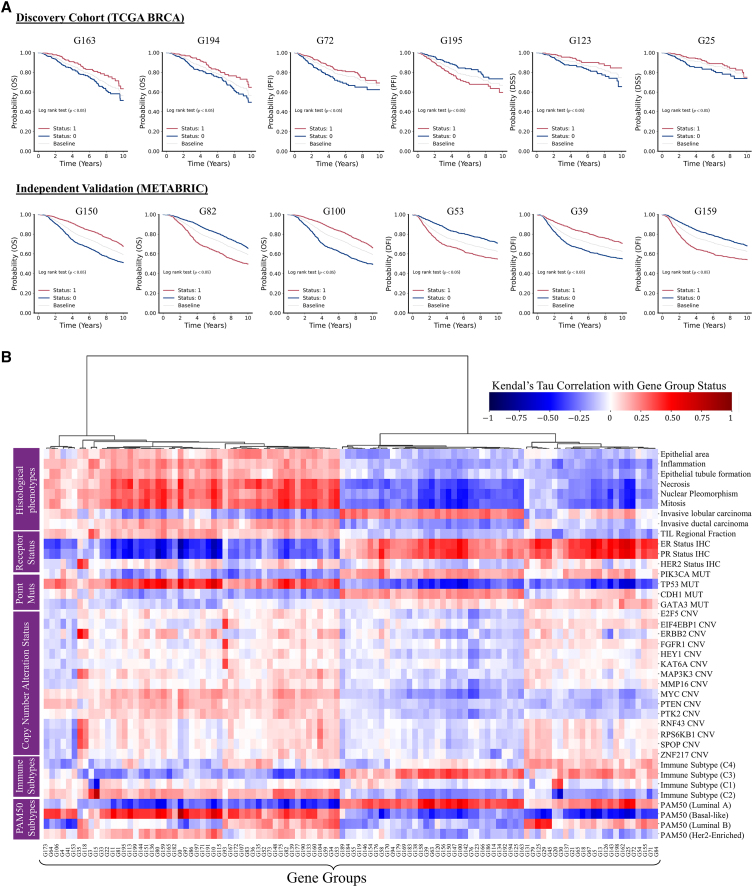
Clinical and pathological significance of gene groups binary status (A) KM curves showing stratification of patients into high- and low-risk groups based on gene group binary status across the discovery cohort and the independent test set. The plots shows that the binary status of several gene groups is associated (log-rank test false discovery rate-corrected p value <0.05) with 10-year censored OS, PFI, DSS, and DFI. As baseline, we also show the survival curve of all patients in the cohort without stratification. (B) Association of gene groups with histological phenotypes, receptor status, genes point MUT status and CNA status, and also immune and PAM50 molecular subtypes. Gene groups are shown along the x axis, and histological phenotypes and other clinical markers are shown along y axis. Red and blue colors indicate the degree of association between gene groups status and a specific histopathological phenotype or clinical marker. Dark red color shows strong positive correlation while strong negative correlation is shown using a dark blue color. CNV, copy number variations.

We found the binary status of several gene groups associated with overall survival (OS), disease-specific survival (DSS), progression-free survival (PFI), and relapse-free survival (DFI) of patients. Figure 3A shows the Kaplan-Meier (KM) survival curves (DSS, PFI, DFI, and OS) illustrating stratification of patients based on their gene group status across the discovery cohort (TCGA) and independent validation set (METABRIC). The KM curves show that patients can be stratified into high- and low-risk groups based on the binary status of a number of gene groups with statistical significance (log rank test false discovery rate-corrected p value <0.05), even though they have been obtained without any direct use of survival information For example, patients with G72 = 1 have a higher survival probability compared with G72 = 0 patients. Overall, in the discovery cohort, there are 25, 3, and 2 gene groups showing significant risk stratification for DSS, OS, and PFI, respectively. Similarly, in the independent validation set, there are 124 and 136 gene groups with significant risk stratification for DFI and OS, respectively. An exhaustive list of all gene groups with significant risk stratification is provided in the Supplemental information.

#### Association between gene groups and breast cancer receptor status

We found the status of several gene groups associated with estrogen receptor (ER), progesterone receptor (PR), and Her2 status, as can be seen in Figure 3B. For example, from the figure a strong positive association of G25 status with ER (Kendall-tau correlation coefficient $\rho_{\tau }=$ 0.68 and p< 0.01) and PR ($\rho_{\tau }=$ 0.58 and p< 0.01) status can be seen. Similarly, we found G35 and G118 status strongly positively associated with her2 status, as shown in Figure 3. Apart from discovery cohort, we found consistent association pattern between patients’ gene groups status and receptor status in the independent validation cohorts can also be seen in Figure S1.

#### Association with PAM50 molecular subtypes and immune subtypes

We found the status of several gene groups associated with PAM50 molecular subtypes, as can be seen in Figure 3B. For example, from the figure, strong positive and negative associations of G25 status can be seen with Luminal A and basal-like subtypes, respectively. Since G25 status has also shown strong association with ER and PR status its correlation with Luminal A (ER positive, PR positive, and Her2 negative) and basal-like (triple negative) subtype is not surprising, but highlights the versatility of gene group definitions. Similarly, similar associations between patients’ G25 status and these molecular subtypes can also be seen in the independent validation cohorts (see Figure S3).

Apart from PAM50 subtypes, we found the status of several gene groups associated with immune subtypes (C1, C2, C3, and C4) defined by Thorsson et al.^24^ as shown in Figure 3B. For example, from Figure 3B, a strong association of G15 can be seen with C2 ($\rho_{\tau }=$ 0.72, p< 0.01) and C1 ($\rho_{\tau }=$ −0.48, p< 0.01) and C3 ($\rho_{\tau }=$ −0.31, p< 0.01). This association is expected as majority of G15 genes (*IFIT3*, *OAS3*, *IFI44L*, etc.) are interferon-regulated genes that play a role in the innate immune response and antiviral defense.^25^

#### Association with mutations in cancer genes

We found the status of several gene groups associated with gene point mutation (MUT) status and copy number alteration (CNA) status as shown in Figure 3B. For example, from Figure 3B, a strong negative correlation of G25 status with TP53 MUT status ($\rho_{\tau }=$ −0.59, p< 0.01) and *MYC* CNA status ($\rho_{\tau }=$ −0.26, p< 0.01) can be seen. Similarly, the status of several other gene groups can be seen as positively or negatively associated with MUT status (e.g., *CDH1*, *GATA3*, and *PIK3A*) and CNA status (e.g., *ERBB2*, *PK2*, *HEY1*, *FGFR*, and *F2F2*) of genes. Furthermore, we found consistent association pattern between patients’ gene groups status and gene alteration status in CPTAC-BRCA and METABRIC cohorts, as shown in Figure S3.

#### Association of gene groups with pathologist-assigned histological phenotypes

We found gene group status to be associated with routine clinical features such as histological types (invasive lobular and ductal carcinoma), histological grade (mitotic count, nuclear pleomorphism, and epithelial tubule formation)^26^ and the spatial fraction of tumor regions with tumor-infiltrating lymphocyte (TIL regional fraction)^27^ as shown in Figure 3B. For example, from the figure, a positive correlation between G3 status and TIL regional fraction can be seen. Similarly, the status of G25 can be seen negatively associated with mitosis, necrosis, nuclear pleomorphism, inflammation, and tumor grade, whereas it can be seen positively associated with invasive lobular carcinoma. This analysis shows that gene group status can be associated with pathologist-assigned histological phenotypes.

### Prediction of gene groups from histological imaging

To explore the association between phenotypic information contained in the WSI and the expression status of a set of genes in a certain gene group we have developed a deep learning-based multi-task graph neural network pipeline (${SlideGraph}^{\infty }$) that takes a WSI as input and predicts the status of 200 gene groups simultaneously. The workflow of the proposed approach is shown in Figure 1B. It builds on our previous work that can model a WSI as a graph to capture histological context, but has been significantly expanded and improved.^28^ Additionally, we also compared ${SlideGraph}^{\infty }$ predictive performance with two other weakly supervised algorithms, namely, clustering-constrained attention multiple instance learning (CLAM)^29^ and Attention-MIL.^30^

#### Quantitative results of gene groups status prediction

**Figure 4:**
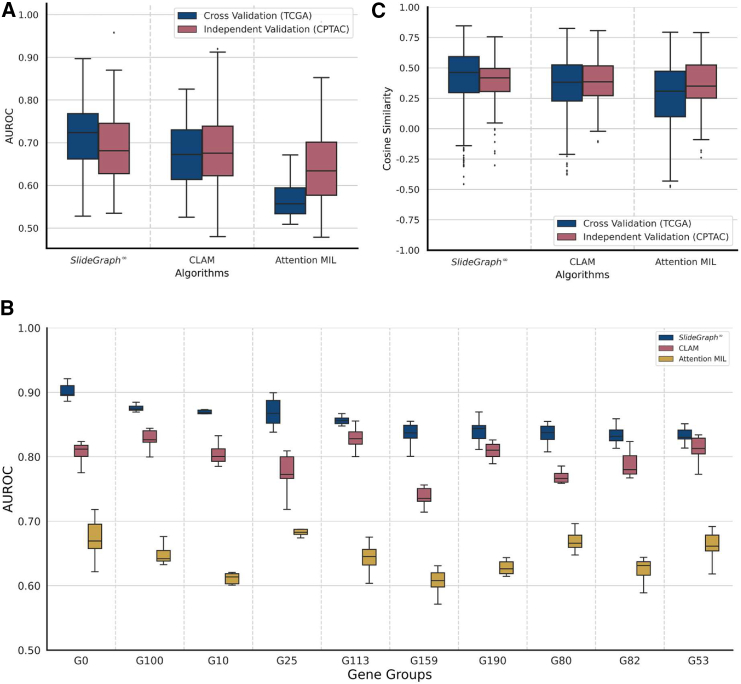
Quantitative result of ${SlideGraph}^{\infty }$ and its performance benchmark with CLAM and Attention-MIL (A) Boxplots showing AUROC distribution of ${SlideGraph}^{\infty }$ and other benchmarked methods over the cross-validation and independent validation cohorts. Each box shows the AUROC distribution at which the status of different gene groups is predicted from WSIs. ${SlideGraph}^{\infty }$ outperform (paired-sample t test, p < 0.05) CLAM and Attention-MIL on the cross-validation cohort, whereas on the external validation cohort its performance is not non-inferior to CLAM and better than Attention-MIL (p < 0.05). (B) Boxplot showing AUROC distribution of ${SlideGraph}^{\infty }$, CLAM and Attention-MIL for a selected number of gene groups across 1,000 bootstrap runs. (C) Plots showing alignment between patients true and predicted gene expression state in terms of cosine similarity for ${SlideGraph}^{\infty }$ and other benchmarked methods over the cross-validation and independent validation cohorts. Each box shows the distribution of cosine similarity between the true and predicted gene expression state of patients in the cross-validation cohort and independent validation set. ${SlideGraph}^{\infty }$ outperform (paired-sample t test, p < 0.05) CLAM and Attention-MIL on the cross-validation cohort, whereas on the external validation cohort its performance is not non-inferior to CLAM and better than Attention-MIL (p < 0.05).

Our predictive analysis shows that the binary status of a significant number of gene groups can be predicted from histology images with a high area under the receiver operating characteristic curve (AUROC) in both cross-validation and independent validation cohorts, as shown in Figure 4A. Furthermore, the proposed approach performs significantly better (paired-sample t test, p < 0.05) than both CLAM and Attention-MIL over the cross-validation cohort (TCGA-BRCA), whereas, for the external validation cohort (CPTAC-BRCA) the performance of the proposed approach is non-inferior to CLAM and significantly better than Attention-MIL. For a selected number of gene groups, we showed the AUROC distribution of ${SlideGraph}^{\infty }$, CLAM and Attention-MIL across 1,000 bootstrap runs in Figure 4B. From the boxplots, it can be seen that ${SlideGraph}^{\infty }$ predicts the status of these gene groups with a high median AUROC compared with CLAM and Attention-MIL.

To analyze the degree to which the complete gene expression profile of a patient can be predicted from imaging alone, Figure 4C displays distribution of patient-wise cosine similarity between histology image-based inferred gene expression state and true gene expression state. The similarity score shows moderate alignment between the true and predicted gene expression states of each patient (median cosine similarity 0.46 and 0.42) across both cross-validation and independent validation cohorts. Of particular interest are patients whose alignment score is either very high or very low. Some example WSI thumbnails of patients whose expression state is best or poorly predicted from histological imaging are shown in Table S2. These results point to the fact that, although the status of certain groups can be predicted with high accuracy, it is not possible to fully characterize the overall gene expression state of most patients from histological imaging alone. This result is expected due to both technical and underlying biological reasons. For example, histological imaging and gene expression analysis are carried out on different tissue sections and the latter uses “bulk” tissue. Furthermore, not all gene expression changes will have a phenotypic effect that can be observed in a WSI, which in turn allows predictive modeling as illustrated in Figure S4. Moreover, gene expression changes are not the sole determinants of phenotypic changes as other factors, such as protein expression and post-translational modifications, also play a key part. This result also shows that both whole slide imaging and gene expression analysis carry complementary value in understanding disease mechanisms.

#### Spatial profiling and histological phenotypes of gene groups

**Figure 5:**
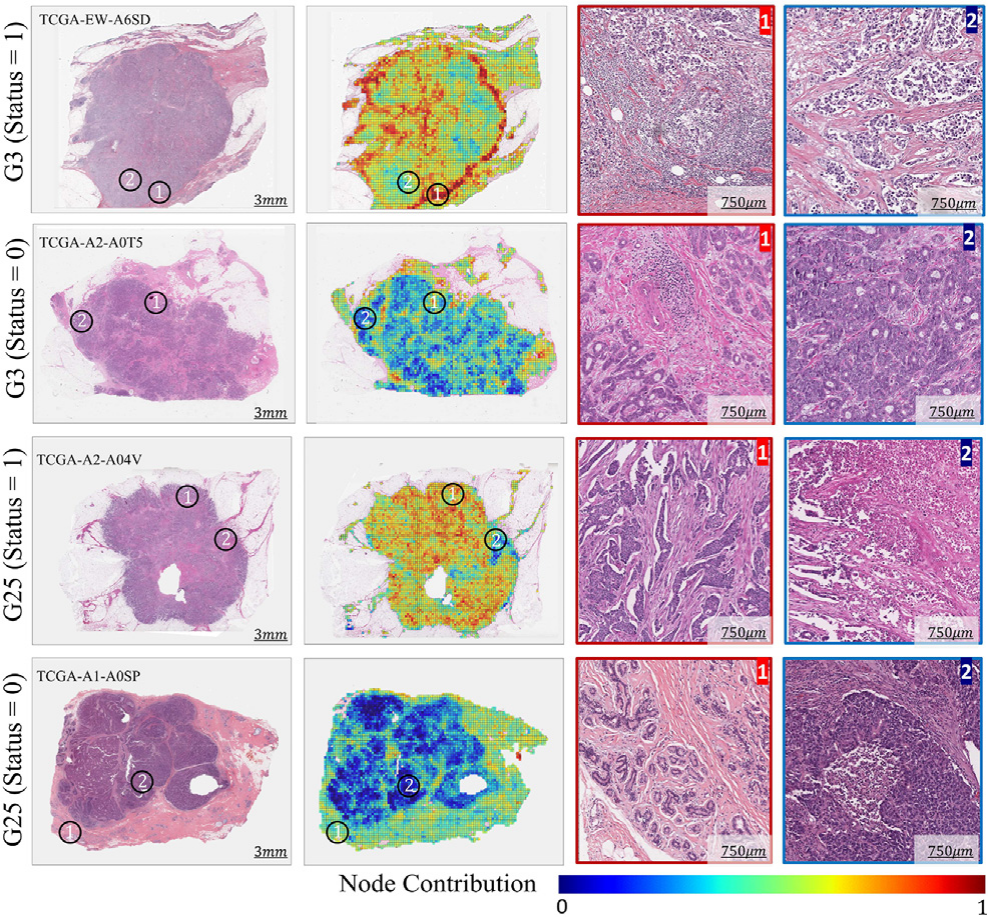
Spatial profiling of gene groups status Spatial profiling of G3 and G25 is displayed through example WSIs and heatmaps. The heatmaps use pseudo colors (bluish to red) to highlight the spatially resolved contribution of patches to the predicted expression state, with bluish and redder color indicating highly contributing status = 0 and status = 1 regions, respectively. From WSIs we extracted magnified version of highly contributing status = 0 and status = 1 regions (ROIs) outlined by red and blue, respectively. The black circles highlight regions of WSIs from which ROIs were extracted. For an interactive visualization, please see tiademos.dcs.warwick.ac.uk/bokeh_app?demo = HiGGsXplore.

The proposed graph neural network can map WSI-level predictions of a gene group to spatially localized regions or nodes in the input image. This enables the profiling of local histological patterns linked to gene groups based on their node-level predictions. Figure 5 shows the spatial profiling of gene groups (G3 and G25 as examples) by visualizing node-level prediction scores from ${SlideGraph}^{\infty }$. For both gene groups, an example WSI with its corresponding heatmap highlighting node level prediction score is shown against binary status 0 and 1. The heatmap highlights the spatially resolved contribution of different regions of the WSI toward the expression status of a certain gene group being 0 or 1. More specifically, regions highlighted in a deeper red color are indicative of an association with status = 1, whereas regions highlighted in a bluish color are indicative of an association with status = 0 of a particular gene group. It is interesting to note that a given gene group exhibits significant variation in prediction scores across different regions of the image, which can be linked to the spatial diversity of localized gene expression patterns throughout the tissue. The localized predictions for other gene groups can be viewed in the HiGGsXplore portal online.

Using node-level prediction scores as a guide, we extracted some regions of interest (ROIs) associated with G3 and G25 status = 0 and 1 from their corresponding WSI, as shown in Figure 5. ROIs representative of G3 = 1 have a relatively high proportion of inflammatory cells compared with G3 = 0 ROIs, where tumor cells seem to be more pleomorphic. Additionally, for the patient with G3 (status = 1), the invasive margin of the tumor, which has a higher density of inflammatory cells, is shown to be correlated with G3 status = 1. Given that G3 status is associated with TIL regional fraction (see Figure 3) and immune response-related processes and pathways (see the Supplementary file on biological significance), TIL is the likely histological phenotype associated with G3 (status = 1).

Regarding G25, tubule formation and normal lobule can be seen in ROIs representative of G25 (status = 1), whereas, in ROIs indicative of G25 (status = 0) the obvious features are necrosis and more pleomorphic tumor cells. For the patient with G25 (status = 1), regions of the WSI with tubule formation are highlighted as illustrated in the ROI. However, for patients with G25 (status = 0) tissue regions with normal lobule received higher score since there was no tissue area with tubule formation. The highlighted spatially resolved histological patterns are concordant with their corresponding enriched cancer hallmark processes (estrogen response, immune response, and p53 signaling) and biological pathways.

#### Mining differential histological patterns associated with each gene group

**Figure 6:**
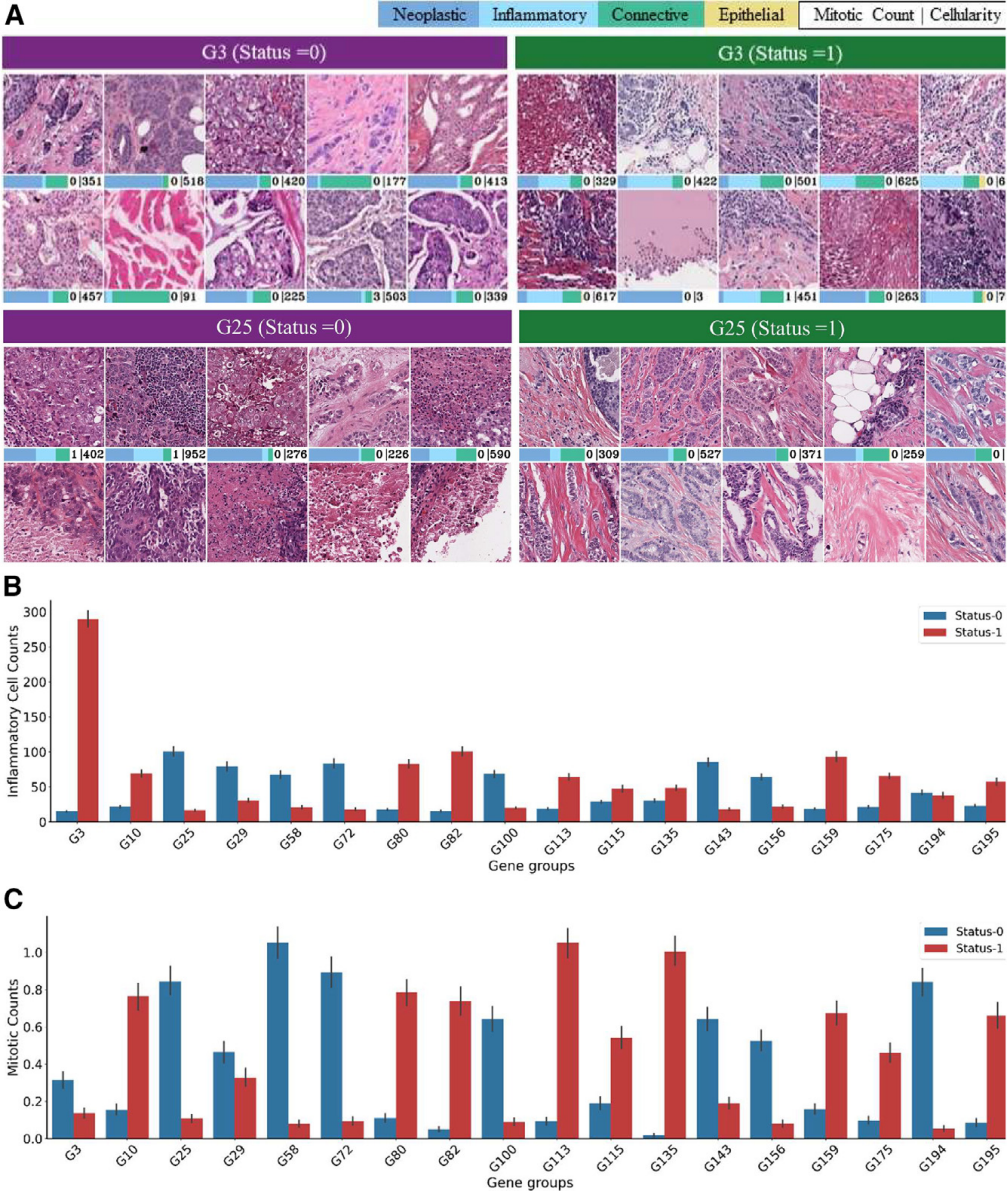
Histological patterns associated with gene groups (A) Representative patches of G25 and G3 statuses 1 and 0 are shown. The bar below the patches shows patch level cellular composition, mitotic counts, and cellularity. (B) Gene groups status (0 and 1) association with patch-level Inflammatory cell counts is shown. (C) Gene groups status (0 and 1) association with patch-level mitotic cell counts is shown.

To explore the association between visual patterns contained in WSIs and gene groups status, we identified 25 exemplar patches for each status (0 and 1) of a certain gene group. For these patches, we also computed the cellular composition (counts of neoplastic, inflammatory, connective, and epithelial cells), overall cellularity, and mitotic counts. Figure 6A shows 10 of 25 representative patches for each of G3 and G25 status = 0 and status = 1. The main difference between G3 = 0 and G3 = 1 patches, as seen in Figure 6, is the presence of lymphoid infiltrate and tumor cellularity. More specifically, G3 = 1 patches have more inflammatory cells and fewer neoplastic cells, whereas the opposite is true for G3 = 0 patches. Additionally, G3 = 0 patches have a relatively higher number of mitotic counts compared with G3 = 1. Regarding G25, the striking difference between G25 = 0 and G25 = 1 patches is the presence of tubule formation (row 2 patches 2 and 3, row 2 images 2 and 3) in the tumor area. As G25 status correlates positively with ER and PR status (see Figure 3B) and previous study has also found ER- and PR-positive cancers enriched in tubule formation,^31^ tubule formation could be the histological phenotype associated with G25 = 1. In contrast, G25 = 0 patches have more pleomorphic sheets of cells and areas of necrosis (row 1 images 1 and 3, row 2 images 1 and 2). This pattern agrees with the histopathological phenotypes we observed in Figures 3B and 5. Finally, G25 = 1 patches show higher mitotic and inflammatory cell counts compared with G25 = 0 patches. Though we are not using any histopathological annotations in training, the predictive model has identified relevant morphometric patterns in an automated manner.

Apart from G25 and G3, we found patch-level inflammatory cell counts and mitotic counts significantly associated (Wilcoxon test p<0.01) with the binary status of several other gene groups, as shown in Figures 6B and 6C.

### Image-based predicted gene group statuses provide latent space representation for downstream predictive modeling

**Figure 7:**
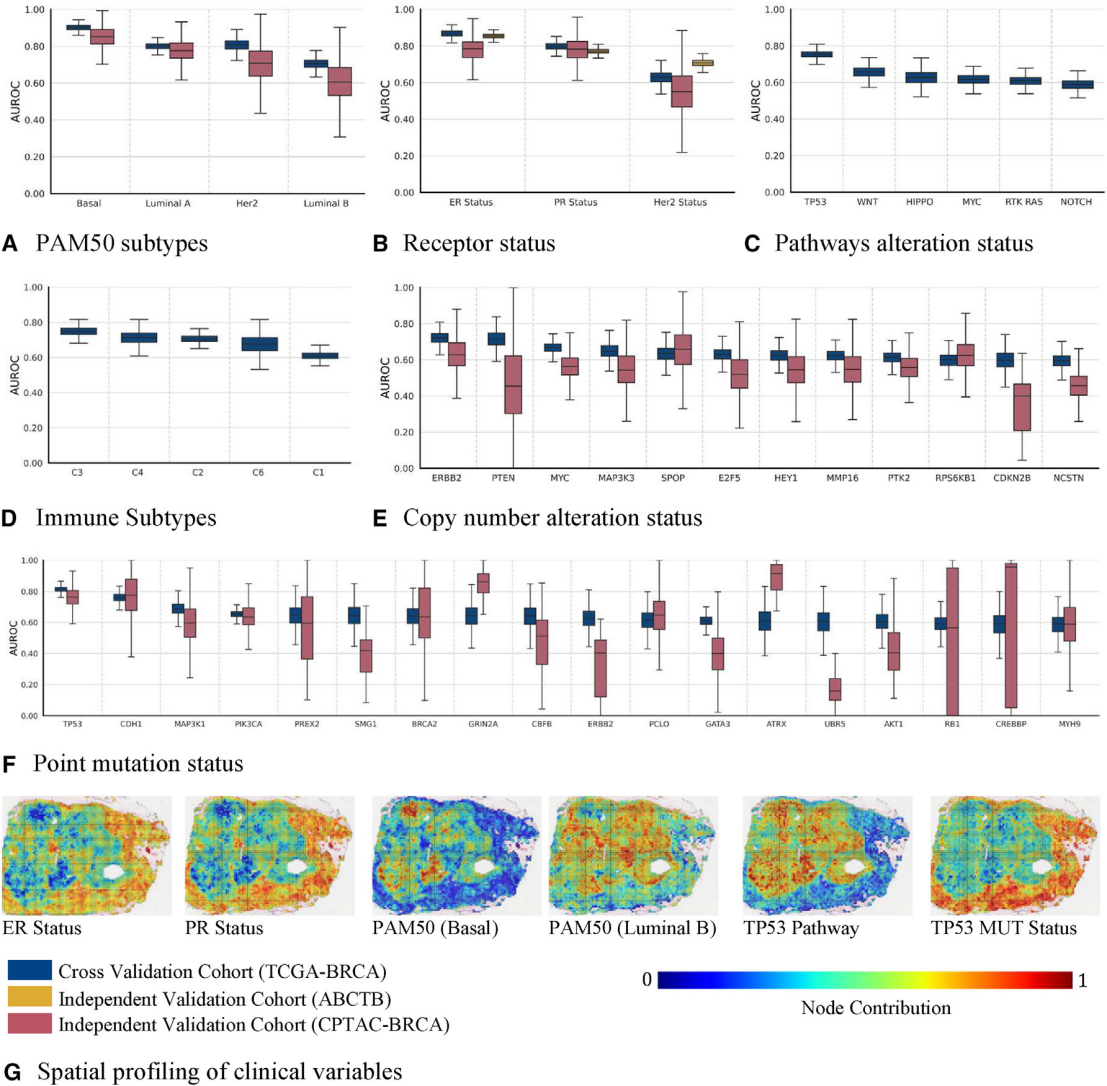
Implication of image-based predicted gene group statuses for downstream predictive modeling (A–F) Prediction of (A) receptor status, (B) PAM50 molecular subtypes, (C) Immune subtypes, (D) pathways alteration status, (E) driver genes CNA status, and (F) point MUT status from image-based predicted gene groups status. Each box in the figure shows the AUROC distribution at which a clinical variable is predicted from image-based predicted gene group status across 1,000 bootstrap runs. Boxes shown in dark blue displays cross-validation AUROC, while the ones shown in light red and yellow show model AUROCs over independent validation cohorts, CPTAC and ABCTB, respectively. (G) Spatial profiling of some routine clinical variables is shown using example heatmaps. The heatmaps use pseudo colors (bluish to red) to highlight the spatially resolved contribution of patches to status = 0 and 1 of a certain clinical variable, with bluish color indicating highly contributing status = 0 regions and red color indicating highly contributing status = 1 regions.

Gene expression groups allow us to capture the gene expression profile of a given patient in terms of 200 gene status variables and their prediction through a machine learning model allows us to map histological patterns to these gene groups. However, the predicted statuses of gene groups can also be used as a compressed latent space representation (LSR) for predictive modeling of other histologically important clinical variables. Figures 7A–7F show the predictability of clinical variables based on the predicted gene group statuses as latent variables using a simple linear classifier. From the boxplots, the LSR can predict patient PAM50 subtype (basal, luminal A, luminal B, and Her2), and receptor status (ER, PR, and Her2), with high AUROC across all datasets. For example, the (ER, PR, and Her2 status) of patients in the TCGA-BRCA and ABCTB cohort can be predicted with a median AUROC values of (0.88, 0.78, and 0.60) and (0.85, 0.77, and 0.70), respectively. Similarly, we found the LSR predictive of signaling pathways alteration status (e.g., AUROC of 0.75 for the TP53 pathway),^32^ immune subtype, and gene MUT status and CNA status. For example, the LSR can predict the *TP53* point MUT status of patients in TCGA-BRCA and CPTAC-BRCA cohort with median AUROC values of 0.82 and 0.76, respectively. Similarly, LSR can also predict *ERRB2* CNA status of patients in both cohorts with AUROC values of 0.71 and 0.62, respectively. Last, we have presented some example heatmaps (Figure 7G) illustrating the spatial profiling of some of these clinical variables (i.e., ER and PR status, PAM50 and basal subtypes, and TP53 MUT and pathway alteration status). From the heatmaps, similar regions can be seen highlighted for the status of ER and PR, while opposite regions for basal subtypes (ER, PR, and Her2 negative).

### Clinical and therapeutic significance of best-predicted gene groups

**Figure 8:**
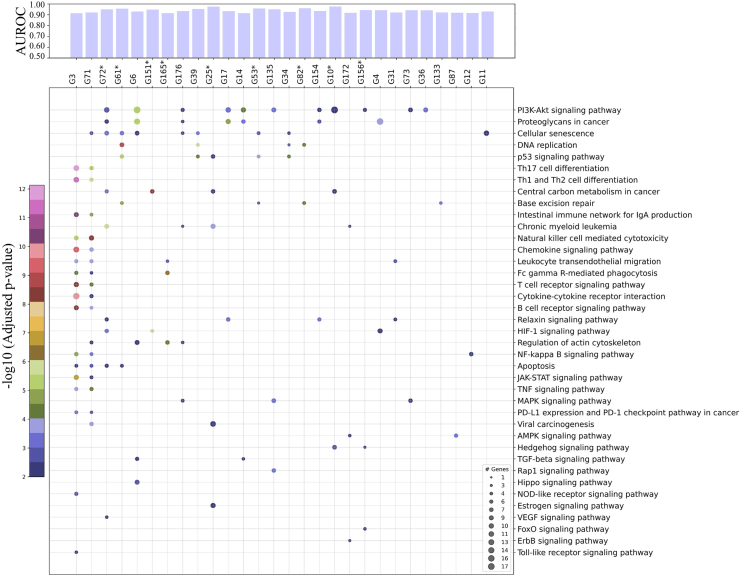
Clinical and therapeutic significance of best predicted gene group The scatterplot shows association of gene groups with biological pathways with gene group shown along x axis (one per column) and corresponding enriched pathways on y axis (one per row). The size of scatter dot shows the number of genes from a particular gene group that has shown significant association (false discovery rate-adjusted p value <0.01) with a certain biological pathway. In the plot the p value is represented by the color of scatter dots. The top bar plot shows the prediction accuracy (AUROC) at which the status of these gene groups are predicted from histology images. Gene groups that show significant association with DSS are annotated with an ∗ next to the gene group name.

We found that gene groups predicted with high accuracy (AUROC ≥ 0.75) from imaging are significantly associated with DSS, biological pathways and hallmark processes. All gene groups associated with DSS are predicted with high accuracy from imaging. Besides this, some interesting biological pathways (see Figure 8) and cancer hallmark processes (see Figure S5) can also be inferred from images based predicted gene groups that can be useful in histology image-based therapeutic decision-making (e.g., drugs targeting the PI3K-Akt pathway in breast cancer).^33^

## Discussion

We performed histological and molecular characterization of breast cancer patients using a purely data-driven approach. Highlighting the limitations of previous methods that predict the expression level of individual genes from histology image, we have shown that significant co-dependencies of different genes across samples (see Figure 2B) compromises the ability of deep learning models to identify individual gene-level genotype to phenotype mapping. To tackle this, we first grouped genes whose expression patterns are significantly dependent and covarying across samples and then proposed a multi-output graph-based deep learning pipeline (${SlideGraph}^{\infty }$) that predicts both WSI-level and spatially resolved expression status of these gene groups in an end-to-end manner. Using the proposed computational pathology workflow, we demonstrated that the status of a significant number of gene groups can be predicted with high accuracy from imaging. This not only overcomes the limitations of existing image-based gene expression prediction models, but provides opportunities to gain biological insights from imaging directly. Finally, we showed that histopathological patterns associated with several gene groups in terms of cellular composition, mitotic counts, and exemplar patches can be identified using the proposed computational pathology pipeline.

A potential advantage of the employed gene grouping approach is the interpretability of gene groups. The method allows a compact representation of a patient’s gene expression state (200 binary latent variables) without losing interpretability, which is crucial in this context, as it provides insight into biological processes and underlying PPIs and PDIs that can motivate new therapies. Through GSEA, we found genes from several gene groups associated with cancer hallmark processes (e.g., epithelial-mesenchymal transition, inflammatory response, estrogen early and late response, mTORC1 signaling, Myc targets, p53 signaling, KRAS up- and down-signaling) and biological pathways (e.g., inflammatory response, PD-L1 expression and PD-1 checkpoint, cancer immunotherapy by PD-blockade and epidermal growth factor/epidermal growth factor receptor signaling). Additionally, we have shown that genes in a certain gene group are enriched for PPI (see Figure 2D), which can provide some insights and possibility new ideas for agents that, event if they have traditionally not been shown to work, might be worth reconsidering.^34^ This could render gene groups and their status a useful guide in analyzing group of patients who have traditionally exhibited limited or no response to therapy, thereby generating ideas for new approaches.

Another important observation regarding gene grouping is that, although the gene groups are defined in a completely data-driven manner without any intelligent selection, still they carry significant clinical meaning in terms of association with survival (OS, DSS, and PFS), routine clinical biomarkers (ER, PR, and Her2 status), driver genes MUT status, and previously defined PAM50 and immune subtypes. For example, the majority of genes that has been previously found associated with immune response^24^ are grouped together (e.g., G3 and G15). This highlights the versatility of gene groups to be used as markers for immune activity, as well as existing molecular subtyping. Apart from this, we found the binary status of several gene groups associated with histopathological annotations, which enable direct genotypic-to-phenotype mapping. Additionally, this genotype to phenotype link can further be validated using GSEA and specialized immunohistochemical (IHC) staining. These results not only validate the clinicopathological significance of these gene groups, but also provide a broader picture of an individual tumor by illuminating the interplay between patient gene expression state and several other clinical variables of interest.

An important feature of the proposed approach for mapping patient gene expression status with morphometric patterns contained in the WSIs is its reliability and explainability. Localized histological patterns identified by ${SlideGraph}^{\infty }$ can be explained in terms of enriched hallmark process, biological pathways, and underlying PPI, as well as through specialized IHC staining and genome sequencing. For example, we found genes from G3 enriched for several immune-related biological processes and pathways, including PD-L1 expression and PD-1 checkpoint pathway, which in histological images we found to be associated with a high proportion of TILs. As G3 status is defined by the expression level of several immune-related genes (e.g., *IL2*, *CD27*, *CCL5*, *PD-1*, and *PD-L2*),^24^^,^^35^ a high proportion of TILs might be the histological phenotypes associated with G3 = 1. Although the observation is interesting, further validation is needed using IHC data. After validation, this could be useful in therapeutic decision-making coupled with other information. Regarding G25, we found tubule formation in the majority of G25 = 1 representative patches, which was consistent with IHC ER and PR status and also the associated cancer hallmark process (estrogen signaling). In contrast, G25 = 0 patches have more pleomorphic sheets of cells several with area of necrosis, which is again concordant with their association with pathologist-assigned phenotypes (necrosis and nuclear pleomorphism), TP53 MUT status, and the p53 signaling pathway. This shows that the proposed deep learning pipeline has identified relevant spatially resolved histological patterns associated with the status of gene groups in an automated manner.

Image-based prediction of gene expression state will open doors of gaining biological insights from imaging directly and is expected to be advantageous in both cancer research and clinical setup. In cancer research, the proposed approach can be used for studying the interplay between gene expression and histopathological phenotypes. Additionally, it can also be used by pharmaceutical industries in their drug discovery pipeline when they study the response of lead compounds in early phase trials. In clinical setup, it will allow cost-effective precision diagnostic from imaging data alone. The proposed computational pathology pipeline not only predicts patient gene expression but also provides a detailed insight in terms of patient survival (OS, DFS, and PFS), possible up or downregulated biological processes and their underlying PPI, possibly mutated or copy-altered genes, and information about ER, PR, and HER2 status, PAM50, and immune subtypes. These types of analysis will provide a more detailed insight into an individual tumor in a cost-effective way. Although by using the proposed approach we managed to predict the expression status of several gene groups with high accuracy and we extensively validated the results on multiple independent validation cohorts, further extensive validation on a large multi-centric dataset is needed before entering clinical practice.

### Limitations of the study

While we validated the association between localized histological patterns and gene groups status, there is still a need for large-scale validation of localized prediction using spatially resolved mRNA expression data. Second, we inferred protein-protein, protein-drug, and pathway activation states using gene groups definition; however, we have not validated these findings at the functional protein level. Furthermore, most protein pathways can be regulated by post-translational modifications like phosphorylation, which cannot be readily predicted from mRNA or protein expression data. Therefore, we assert that extensive validation is needed, not only to validate the PPIs and PDIs at the functional protein level, but also the activation signatures of protein pathways.

## STAR★Methods

### Key resources table

**Table:** 

REAGENT or RESOURCE	SOURCE	IDENTIFIER
**Deposited data**		

Whole Slide Images (TCGA)	The Cancer Genome Atlas	RRID:SCR_003193
Whole Slide Images (CPTAC)	The Cancer Imaging Archive	https://www.cancerimagingarchive.net/collections
Molecular Data (TCGA)	National Cancer Institute	RRID:SCR_003193
CPTAC Proteogenomic Data	cBioPortal	https://www.cell.com/cancer-cell/fulltext/S1535-6108(23)00219-2
Whole Slide Images and Receptor Status (ABCTB)	Australian Breast Cancer Tissue Bank	https://doi.org/10.1089/bio.2014.0055

**Software and algorithms**		

CorEx	https://github.com/gregversteeg/bio_corex	N/A
tiatoolbox	https://github.com/TissueImageAnalytics/tiatoolbox/	v1.4
ALBRT	https://github.com/engrodawood/ALBRT	v1.4
STITCH	http://stitch.embl.de/	STITCH 5
torch geometric	https://pytorch-geometric.readthedocs.io/en/latest/install/installation.html	v2.2
lifelines	https://lifelines.readthedocs.io/en/latest/	v0.3.0
PyTorch (2.0)	https://pytorch.org/	RRID:SCR_018536
Scipy (1.6.2)	http://www.scipy.org/	RRID:SCR_008058
NumPy (1.23.5)	http://www.numpy.org/	RRID:SCR_008633
Pandas (1.5.3)	https://pandas.pydata.org/	RRID:SCR_018214
Matplotlib (3.3.4)	https://matplotlib.org/	RRID:SCR_008624
HiGGsXplore	This paper	https://doi.org/10.5281/zenodo.10053647.

### Resource availability

#### Lead contact

Further information and requests regarding this manuscript and experimental code should be directed to and fulfilled by the lead contact, Fayyaz ul Amir Afsar Minhas (Fayyaz.Minhas@warwick.ac.uk).

#### Materials availability

This study did not generate new materials.

#### Data and code availability

• Whole slides images (WSIs) of all TCGA-BRCA patients used in the study can be downloaded from NIH Genomic Data Common Portal at this link: https://portal.gdc.cancer.gov/ with the manifest details included in the supplemental information.
• The genomic data and clinical data of TCGA-BRCA, METABRIC and CPTAC-BRCA cohort can be downloaded from cBioPortal https://www.cbioportal.org/. Patient ids and field details are included in the supplemental information.
• ABCTB data and images were obtained from the Australian Breast Cancer Tissue Bank. The lead author can be contacted to facilitate access to the images and receptor status of these patients.
• The deep learning model was developed using PyTorch Geometric library and TIAToolbox.^36^ Code and documentation of all python scripts used in the study can be found at: https://doi.org/10.5281/zenodo.10053647.
• Any additional information required to reanalyze the data reported in this work paper is available from the lead contact upon request.

### Experimental model and study participant details

#### Patient cohorts

Biomedical and Scientific Research Ethics Committee (BSREC) University of Warwick Approved the study under application ID BSREC 16/21–22. All samples used in the study were obtained with research consent and ethics approvals as indicated in the consent and ethics statements for TCGA (The Cancer Genome Atlas),^37^^,^^38^ CPTAC (Clinical Proteomic Analysis Consortium),^39^ METABRIC (Molecular Taxonomy of Breast Cancer International Consortium)^40^^,^^41^ and ABCTB (Australian Breast Cancer Tissue Bank)^36^ given in the references provided herewith.

In this study we used data from four breast cancer cohorts namely TCGA-BRCA (TCGA breast cancer cohort),^37^^,^^38^ CPTAC-BRCA (CPTAC breast cancer cohort),^40^^,^^41^ METABRIC^40^^,^^41^ and ABCTB.^36^ For TCGA-BRCA (n=1082), CPTAC-BRCA (n=122), and METABRIC (n=1980) cohorts we collected mRNA expression, gene mutation status (MUT status), copy number alterations status (CNA status), and available clinical data from cBioportal.^42^^,^^43^ For TCGA-BRCA cohort we collected 1,133 Whole Slide Images (WSIs) of Formalin-Fixed Paraffin-Embedded (FFPE) Hematoxylin and Eosin (H&E) stained tissue section of 1,062 patients from the Cancer Genome Atlas (TCGA).^37^^,^^38^ For patients with multiple slides, we selected the one with best visual quality. Additionally for robust analysis, we ignored WSIs with missing baseline resolution information. The survival data for TCGA-BRCA cohort were used from Pan-Cancer Clinical Data Resource (TCGA-CDR).^44^ For the CPTAC cohort we downloaded 653 WSIs of 122 patients from The Cancer Imaging Archive (TCIA).^45^ Finally, within ABCTB cohort, we have access to WSIs and receptor status (ER, PR and Her2 status) information, for a total of 2,303 patients.

### Method details

#### mRNA expression data preprocessing

We first converted the gene expression data into log2 normalized *Z* score values. Afterward, using expression data of TCGA-BRCA cohort we selected a set of 5,596 genes having high variance in expression across patient samples along with known oncogenes. After gene selection we end up with a matrix of size N×5,676 for TCGA-BRCA, CPTAC-BRCA and METABRIC cohort where N represents the number of patients in the cohort.

#### Data Driven Discovery of gene groups with CorEx

To model associations between expression profile of different genes we used Total Correlation Explanation (CorEx) on the gene expression matrix M of size m×n where m and n are the number of patient samples, and genes, respectively.^46^ As the expression of different genes is significantly inter-dependent and correlated, CorEx allows us to represent the gene expression state of a patient in terms of a small number of binary variables or gene groups that can capture information contained in the expression of all genes of a given patient with minimal loss. CorEx initiates the processes by discovering groups of genes whose expression are significantly inter-dependent. CorEx then assigns binary labels to each gene group directly without any binarization of continuous values by ensuring that the gene group status shows significant mutual information with the expression level of genes in the group. Thus, these binary group statuses “explain away” co-dependence of expression across genes for a given patient without the need of any binarization resulting in a more easily interpretable expression sate. The binary status of each gene group indicates the existence of specific patterns of co-dependent expression across genes. As the outputs are binary, there is no need for any arbitrary threshold selection for binarization or the use of regression. A potential advantage of employing binary status is that it simplifies the learning problem (histology image-based prediction of gene expression state) by reducing the possible patient gene expression profiles from thousands of genes to 200 binary statuses for each patient. For a detailed mathematical formulation underlying CorEx, the interested reader is referred to the CorEx paper.^46^ Given $M_{m\times n}$ as input, the output of CorEx is a matrix $G_{m\times d}$ with each column of G corresponds to a binary latent factor $G_{k}$ (k=1…d with d≪n) so that the mutual information between the expression level of genes is minimized after conditioning on $G_{1},\ldots \text{,}G_{d}$. Akin to “loadings” in principle component analysis (PCA), the definition of each binary latent factor $G_{k}$ is based on mutual information between the expression score of a certain gene and the binary status of $G_{k}$ across patient samples. This allows us to model each of the latent factors as a ranked (by mutual information) collection or group of genes. However, unlike PCA (or other linear or kernelized dimensionality reduction techniques based on covariance), CorEx can capture non-linear statistical relationships and dependencies between input variables (genes) directly due to its use of mutual information (see comparative analysis in^46^). We run the algorithm for 100 iterations on the *Z* score expression of TCGA-BRCA patients for discovering 200 binary latent factors. The number of latent factors were decided based on the TC distribution shown in Figure S6. The distribution demonstrates that the overall TC (sum of TCs of all latent factor) plateaus and approaches zero after selecting 200 latent factors. Therefore, we selected 200 latent factors. The binary statuses of these 200 latent variables define the expression state of a patient, where the binary value of each latent variable is defined by the group of genes whose gene expression patterns are substantially co-dependent across samples as shown in Figure 2B.

##### Analysis of biological and therapeutic significance of gene groups

Hallmark processes and KEGG pathways enrichment for genes in different gene groups were obtained using Enrichr.^47^ In line with previous work,^20^ we selected a maximum of top 400 genes from each gene group whose mutual information is greater than 0.002. We passed the gene set to Enrichr which returns the enriched terms across a selected library (in our case KEGG pathway and MSigDB hallmarks) coupled with their statistical significance (FDR-adjusted p value using Benjamini-Hochberg methods). We used a cutoff value of p<0.01 on the adjusted p value for statistical significance of an enriched term across the selected library. The protein-protein and drug-protein interactions are analyzed using STITCH.^48^

##### Independent validation of the gene group discovery approach

We validated the results of the employed gene group discovery algorithm (CorEx) on two independent validation cohorts (CPTAC-BRCA and METABRIC). More specifically, we inferred the gene groups status of patients in these cohorts using CorEx model trained on mRNA expression data of TCGA-BRCA cohort. We visualized the consistency between patient’s gene group status for a certain gene group and expression levels of set of genes in that group by generating some example heatmaps (see Figure 2). Additionally, we also analyzed the consistency of association between gene group status and histological phenotypes, receptor status, molecular subtypes, gene point mutation and copy number alteration status.

##### Analysis of batch effects and other confounders

In our previous work^49^ we showed that site-specific signatures are manifest in TCGA samples. To ensure that the gene groups obtained from CorEx are not biased toward source sites, we analyzed the degree of predictability of tissue source site of a given sample based on the gene groups assigned to it. The hypothesis is that if the source site of a sample can be predicted with high accuracy or AUROC using its gene group signature then there is a substantial bias in the assignment of groups or the possibility of site-specific batch effects. For this purpose, we trained both linear (SVM) and non-linear (XGBoost) models to predict the site of origin of a sample from its gene group. We evaluated the performance of both SVM and XGBoost using 5-fold cross validation and reported the mean and standard-deviation of these models. If the source sites are predictable with high mean AUROC values, then it signifies that the gene groups might suffer from potential batch effect. However, if the sites are predictable with low AUROC then the gene groups do not suffer from any marked biased or batch effect.

#### WSI analysis pipeline with ${SlideGraph}^{\infty }$

##### Preprocessing of whole slide images

We segment the tissue regions of WSIs using a tissue segmentation model and ignore regions with tissue artifacts (pen-marking, tissue folding, etc.). Each WSI is then tiled into patches of size 512×512 pixels at a spatial resolution of 0.50 microns-per-pixel (MPP). Patches capturing less than 40% of informative tissue area (pixels with intensity higher than 200) are discarded, and the remaining patches (both tumor and non-tumor) are used.

##### WSI-graph construction

A graph =(V,E) is defined by a vertex set V, and an edge set E. The set $V=\left\{v_{i}|i=1,\ldots N\right\}$ defines nodes in a graph (in our case is the set of patches in a WSI) while connectivity between nodes is defined by the edges E. Each node $v_{i}=\left(g_{i},h_{i}\right)$ captures the spatial location ($g_{i}$), and feature representation ($h_{i}$) of a patch in the WSI. We obtain the feature representation $h_{i}\in R^{1024}$ of a patch $x_{i}$ by extracting latent representation from ShuffleNet^44^ pretrained on ImageNet.^50^ The edge set E is obtained by connecting nodes to the neighboring nodes (distance less than 4000 pixels) using Delaunay triangulation. If two nodes $v_{i}$ and $v_{j}$ are connected, then there will be an edge $e_{ij}\in E$.

##### Gene expression state prediction using graph neural network

We pass the graph representation of a WSI through a Graph Neural Network (GNN) for predicting the node-level and WSI-level expression status of all gene groups simultaneously. In this work, we have developed a custom multi-output GNN that predicts the patch-level and WSI-level expression statuses of different gene groups in an end-to-end manner. Node level representation is passed through EdgeConv layers L={1,2,3}. Each EdgeConv layer^51^ updates the representation of each node in the graph by aggregating the information from their neighboring node and generates embedding for successive layers. For a node in layer l at index m the output embedding of EdgeConv layer can mathematically be written as follows:

$$h_{m}^{l}=\sum_{k\in \;ℵ\left(m\right)}H^{l}\;\left(h_{m}^{l-1}∥h_{k}^{l-1}-h_{m}^{l-1}\right)$$

In the above equation $h_{m}^{0}=h_{m}$, ℵ(m) represents the neighboring nodes of m, and $H^{l}$ denote a neural network. EdgeConv operation is trying to combine information of a node $h_{m}^{l}$ and neighboring nodes ℵ(m). Since we are using three EdgeConv layers, each node is expected to capture information from the neighboring nodes that are less than 5-hops apart in the WSI-graph.

For spatial profiling for gene expression groups, the feature representation $h_{m}^{l}$ of a node $v_{m}=\left(g_{j},h_{j}\right)\in V$ is passed as input to a multilayer perceptron $f_{l}\left(v_{m}\right)=f\left(h_{m}^{l}\right)$ for generating node level prediction score which is then aggregated across all layers for getting patch level prediction score for all gene groups.

$$f\left(v_{m}\right)=\sum_{l=0}^{L}f_{l}\left(h_{m}^{l}\right)$$

The WSI-level score for the expression status of all gene groups is obtained by pooling and aggregating node-level prediction scores as follows:

$$F\left(G\right)=\sum_{\forall \;m\in V}f\left(v_{m}\right)$$

The trainable parameters of the EdgeConv layers and node-level classifiers are learned in an end-to-end manner using backpropagation. In a training batch of size N, the model predicted score for k={1…K} binary latent factors are compared with their ground truth value using pairwise ranking loss,^28^ mathematically formulated as follows:

$$L=\sum_{k}\sum_{\left(a,b\right)\in P_{k}}\max\left(0,1-\left(f^{k}\left(X_{a}\right)-f^{k}\left(X_{b}\right)\right)\right)$$

Here $P_{k}=\left\{\left(a,b\right)|y_{a}^{k}>y_{b}^{k},a,b=1\ldots .N\right\}$ is the set of all pair of patients (a, b) where the expression status of patient a is greater than patient b for latent factor k. Minimization of the loss function L(∵) will enforce the model to rank status = 1 patients higher than status = 0 for all latent factors.

##### Training and internal validation of ${SlideGraph}^{\infty }$

We trained and evaluated the performance of ${SlideGraph}^{\infty }$ using 5-fold cross-validation, in which the dataset is subsampled into five 80/20 non-overlapping splits. The model is trained on 80% of the data and 20% data is held out for testing. From the training data we randomly select 10% of the data for parameter tuning and optimization. We train ${SlideGraph}^{\infty }$ on the training set for 300 epochs using the Adam optimizer with an initial learning rate and weight decay of 0.001 and 0.0001, respectively. In each epoch, the training set is sampled into mini-batches of size 8, and the learnable parameters of ${SlideGraph}^{\infty }$ are updated using adaptive momentum-based optimizer. To avoid overfitting, we stop the model training early, if performance over the validation set does not improve for 20 consecutive epochs. During training, we maintain a queue of size 10 for tracking the best models based on their performance over the validation set. More specifically, we insert the model into the queue if the validation loss at epoch n is less than the loss at epoch n−1. For test set inference, we ensemble the prediction score of all the models in the queue by averaging the prediction score and using that as the final prediction. For quantitative performance assessment, we report area under the receiver operating characteristic curve (AUROC) over the test set.

##### Spatial profiling of gene groups and visualization

For a given WSI, the spatially resolved contribution of different tissue regions toward the expression status of a certain gene groups can visualized. We developed an online portal (http://tiademos.dcs.warwick.ac.uk/bokeh_app?demo=HiGGsXplore) which can assist user in spatially resolved cross-linking of genotype-phenotype mapping in terms of these gene groups. More specifically, the portal uses WSI coupled with node level prediction of different gene group and then show the node level prediction in the form of an interactive heatmap. Additionally, the tool can also show different histological features when the user hover over a node in the graph.

##### Identification of histological motifs

To uncover cellular and morphometric patterns associated with the expression status (0, or 1) of a particular gene group we divided patients into two groups (status = 0 and status = 1). For each group, we select 50 patients whose expression statuses are accurately predicted from their WSIs. From each of these WSIs, for patients with status = 1, we extract the highest scoring (based on node-level score) 1% patches, while for status = 0, we extract the lowest scoring patches and then cluster the patches within each group for getting representative patterns. Within each group (status = 0, and 1) we cluster the patches using 25-medoid clustering. After clustering, we get 25 visual patterns (histological motifs) representative of expression status = 0 and status = 1 of a certain gene group.

##### Independent validation of ${SlideGraph}^{\infty }$ predicted gene groups status

We assessed the accuracy of ${SlideGraph}^{\infty }$ predicted gene group statuses on unseen WSIs of patients from CPTAC-BRCA cohort. For patients in the CPTAC-BRCA cohort we first constructed graph representation of their WSIs and then predicted their gene groups status using ${SlideGraph}^{\infty }$ model trained on TCGA-BRCA cohort. For patients with multiple whole slide images in the CPTAC-BRCA cohort we created a bag of graphs and then inferred the gene groups status using the bag of graphs as input to the five models from the cross-validation runs. The final prediction is generated by averaging the prediction of ensemble models. For performance evaluation we reported AUROC values between ${SlideGraph}^{\infty }$ predicted gene groups status and gene expression based inferred gene groups status from CorEx model (trained on TCGA-BRCA cohort) as performance metric. Additionally, we also reported the model accuracy in predicting the overall gene expression state of patients in terms of cosine similarity between image-based predicted status of 200 gene group and gene expression based inferred ground truth.

##### Training and evaluation of downstream predictors

We train separate multi-output perceptron for predicting the receptor status, PAM50 molecular subtypes, Immune subtypes, pathways alteration status, genes point mutation status and copy number alteration status using ${SlideGraph}^{\infty }$ predicted gene groups status as features. The classifier for each downstream task is trained and evaluated using same loss function and training and validation protocol employed for ${SlideGraph}^{\infty }$ training and evaluation. After cross-validation, we get the downstream classifier prediction score for a particular clinical variable of interest for all patients. For performance we subsample 67% of the patients 1,000 times with replacement and compute the AUROC between ground truth and model predicted score.

##### Independent validation of downstream predictors

We assessed the performance of predictors developed on top of ${SlideGraph}^{\infty }$ latent representation for various downstream tasks (PAM50 subtypes, receptor status, gene mutation and copy number alteration status) on two independent validation cohorts (CPTAC-BRCA and ABCTB). For each task we assessed the independent validation performance of the corresponding downstream predictor trained on ${SlideGraph}^{\infty }$ predicted gene groups status and ground truth labels of TCGA-BRCA cohort. The final prediction for each patient is obtained by averaging the prediction of five ensemble downstream predictors.

##### ${SlideGraph}^{\infty }$ comparison with other methods

We compared the predictive performance of ${SlideGraph}^{\infty }$ with CLAM and Attention MIL. As CLAM and Attention MIL support multi-output by design, therefore for predicting the status of each of the 200 gene group we trained a separate model using the same training and validation splits used for ${SlideGraph}^{\infty }$. For reporting quantitative results on internal validation and external validation cohorts we used the experimental setup used for ${SlideGraph}^{\infty }$.

### Quantification and statistical analysis

#### Cellular composition estimations

We estimated the counts of neoplastic, inflammatory, connective, and normal epithelial cells present in a patch using our in-house cellular composition predictor ALBRT.^52^ ALBRT takes a patch of size 256×256 at a spatial resolution of 0.25 MPP and predicts the counts of the types of cells present in it. We extracted patches of size 256×256 at 0.25 MPP using (x, y) of coordinates of 512×512 at 0.50 MPP. For each 512×512 patch, we obtained the cellular composition estimates by aggregating ALBRT-predicted cellular estimates of around 16 256×256 patches. The cellularity was computed by summing the counts of neoplastic, inflammatory, connective, and epithelial cells present in a 512×512 patch.

#### Estimation of mitotic counts

Mitosis detection has been done using the state-of-the-art “mitosis detection: fast and slow” (MDFS) method.^53^ MDFS is a two-stage method where mitotic candidates are first detected using a fully convolutional neural network and then refined by a deeper CNN classifier. Several techniques have been incorporated during the training of the MDFS to make it robust against domain shift problems seen in histology images and generalize better to unseen images. After detecting mitotic figures, we estimate the patch-level mitotic counts by counting all the detected mitoses in the patch.

#### Statistical analysis

For testing statistical significance in Kaplan-Meier analysis, we used log-rank test to measure the difference between the two distributions (i.e., status = 0 and status = 0). As we are iteratively, testing the association of all 200 gene groups with survival, so we corrected the p-value using Benjamini/Hochberg method, and then use a threshold of p≪0.05 for statistical significance. To analyze the difference (in mitotic and inflammatory cell counts) between highly attended patches of status = 0 and 1 of a certain gene group, we used two-sided Wilcoxon rank-sum test between the two distribution and used a threshold of p≪0.05 for statistical significance.

## Supplemental information

Document S1. Figures S1–S6 and Tables S1 and S2

Data S1. Gene Groups status of patients in TCGA-BRCA, CPTAC-BRCA and METABRIC cohorts, related to Figure 2A data-driven discovery of gene groups

Data S2. Gene Set Enrichment Analysis (Hallmark processes, KEGG pathways and Gene Ontology) of genes in all 200 gene groups, related to Figure 2C biological significance of gene groups

Data S3: Pathological significance of binary status of gene groups in terms of association with survival, related to Figure 3A stratification of patients into high and low risk group based on gene group status

Data S4: Quantitative results of ${SlideGraph}^{\infty }$ and its performance benchmark against CLAM and Attention MIL for all 200 gene groups across patients in both cross validation and independent validation cohorts, related to Figure 4) Quantitative result of ${SlideGraph}^{\infty }$ and its performance benchmark with CLAM and Attention MIL

Data S5. Prediction scores of ${SlideGraph}^{\infty }$ for downstream prediction tasks (PAM50 subtypes, receptor status, gene mutation and copy number alteration status, immune subtype and pathways alteration status), related to Figure 7) Implication of Image-based predicted gene group statuses for downstream predictive modeling

Document S2. Article plus supplemental information

## References

[1] Hanahan D. (2022). Hallmarks of Cancer: New Dimensions. Cancer Discov..

[2] Heng Y.J., Lester S.C., Tse G.M., Factor R.E., Allison K.H., Collins L.C., Chen Y.Y., Jensen K.C., Johnson N.B., Jeong J.C. (2017). The molecular basis of breast cancer pathological phenotypes. J. Pathol..

[3] Parker J.S., Mullins M., Cheang M.C.U., Leung S., Voduc D., Vickery T., Davies S., Fauron C., He X., Hu Z. (2009). Supervised Risk Predictor of Breast Cancer Based on Intrinsic Subtypes. J. Clin. Oncol..

[4] Sweeney C., Bernard P.S., Factor R.E., Kwan M.L., Habel L.A., Quesenberry C.P., Shakespear K., Weltzien E.K., Stijleman I.J., Davis C.A. (2014). Intrinsic Subtypes from PAM50 Gene Expression Assay in a Population-Based Breast Cancer Cohort: Differences by Age, Race, and Tumor Characteristics. Cancer Epidemiol. Biomarkers Prev..

[5] Sparano J.A., Paik S. (2008). Development of the 21-gene assay and its application in clinical practice and clinical trials. J. Clin. Oncol..

[6] Buyse M., Loi S., van't Veer L., Viale G., Delorenzi M., Glas A.M., d'Assignies M.S., Bergh J., Lidereau R., Ellis P. (2006). Validation and Clinical Utility of a 70-Gene Prognostic Signature for Women With Node-Negative Breast Cancer. J. Natl. Cancer Inst..

[7] Wang Z., Gerstein M., Snyder M. (2009). RNA-Seq: a revolutionary tool for transcriptomics. Nat. Rev. Genet..

[8] Tang F., Barbacioru C., Wang Y., Nordman E., Lee C., Xu N., Wang X., Bodeau J., Tuch B.B., Siddiqui A. (2009). mRNA-Seq whole-transcriptome analysis of a single cell. Nat. Methods.

[9] Picelli S., Björklund Å.K., Faridani O.R., Sagasser S., Winberg G., Sandberg R. (2013). Smart-seq2 for sensitive full-length transcriptome profiling in single cells. Nat. Methods.

[10] Marx V. (2021). Method of the Year: spatially resolved transcriptomics. Nat. Methods.

[11] Ståhl P.L., Salmén F., Vickovic S., Lundmark A., Navarro J.F., Magnusson J., Giacomello S., Asp M., Westholm J.O., Huss M. (2016). Visualization and analysis of gene expression in tissue sections by spatial transcriptomics. Science.

[12] Merritt C.R., Ong G.T., Church S.E., Barker K., Danaher P., Geiss G., Hoang M., Jung J., Liang Y., McKay-Fleisch J. (2020). Multiplex digital spatial profiling of proteins and RNA in fixed tissue. Nat. Biotechnol..

[13] Dawood M., Branson K., Rajpoot N.M. (2021). Joint European Conference on Machine Learning and Knowledge Discovery in Databases.

[14] He B., Bergenstråhle L., Stenbeck L., Abid A., Andersson A., Borg Å., Maaskola J., Lundeberg J., Zou J. (2020). Integrating spatial gene expression and breast tumour morphology via deep learning. Nat. Biomed. Eng..

[15] Schmauch B., Romagnoni A., Pronier E., Saillard C., Maillé P., Calderaro J., Kamoun A., Sefta M., Toldo S., Zaslavskiy M. (2020). A deep learning model to predict RNA-Seq expression of tumours from whole slide images. Nat. Commun..

[16] Wang Y., Kartasalo K., Weitz P., Ács B., Valkonen M., Larsson C., Ruusuvuori P., Hartman J., Rantalainen M. (2021). Predicting Molecular Phenotypes from Histopathology Images: A Transcriptome-Wide Expression–Morphology Analysis in Breast Cancer. Cancer Res..

[17] Alsaafin A., Safarpoor A., Sikaroudi M., Hipp J.D., Tizhoosh H.R. (2023). Learning to predict RNA sequence expressions from whole slide images with applications for search and classification. Commun. Biol..

[18] Carvunis A.-R., Roth F., Calderwood M., Cusick M., Superti-Furga G., Vidal M. (2013). Handbook of Systems Biology Concepts and Insights.

[19] Paci P., Fiscon G., Conte F., Wang R.-S., Farina L., Loscalzo J. (2021). Gene co-expression in the interactome: moving from correlation toward causation via an integrated approach to disease module discovery. NPJ Syst. Biol. Appl..

[20] Pepke S., Ver Steeg G. (2017). Comprehensive discovery of subsample gene expression components by information explanation: therapeutic implications in cancer. BMC Med. Genomics.

[21] Waldmann T.A. (2006). The biology of interleukin-2 and interleukin-15: implications for cancer therapy and vaccine design. Nat. Rev. Immunol..

[22] Durda P., Sabourin J., Lange E.M., Nalls M.A., Mychaleckyj J.C., Jenny N.S., Li J., Walston J., Harris T.B., Psaty B.M. (2015). Plasma Levels of Soluble Interleukin-2 Receptor α. Arterioscler. Thromb. Vasc. Biol..

[23] Mastropasqua F., Marzano F., Valletti A., Aiello I., Di Tullio G., Morgano A., Liuni S., Ranieri E., Guerrini L., Gasparre G. (2017). TRIM8 restores p53 tumour suppressor function by blunting N-MYC activity in chemo-resistant tumours. Mol. Cancer.

[24] Thorsson V., Gibbs D.L., Brown S.D., Wolf D., Bortone D.S., Ou Yang T.H., Porta-Pardo E., Gao G.F., Plaisier C.L., Eddy J.A. (2018). The Immune Landscape of Cancer. Immunity.

[25] Piera-Velazquez S., Mendoza F.A., Addya S., Pomante D., Jimenez S.A. (2021). Increased expression of interferon regulated and antiviral response genes in CD31+/CD102+ lung microvascular endothelial cells from systemic sclerosis patients with end-stage interstitial lung disease. Clin. Exp. Rheumatol..

[26] Thennavan A., Beca F., Xia Y., Recio S.G., Allison K., Collins L.C., Tse G.M., Chen Y.Y., Schnitt S.J., Hoadley K.A. (2021). Molecular analysis of TCGA breast cancer histologic types. Cell Genom..

[27] Saltz J., Gupta R., Hou L., Kurc T., Singh P., Nguyen V., Samaras D., Shroyer K.R., Zhao T., Batiste R. (2018). Spatial Organization and Molecular Correlation of Tumor-Infiltrating Lymphocytes Using Deep Learning on Pathology Images. Cell Rep..

[28] Lu W., Toss M., Dawood M., Rakha E., Rajpoot N., Minhas F. (2022). SlideGraph+: Whole slide image level graphs to predict HER2 status in breast cancer. Med. Image Anal..

[29] Lu M.Y., Williamson D.F.K., Chen T.Y., Chen R.J., Barbieri M., Mahmood F. (2021). Data-efficient and weakly supervised computational pathology on whole-slide images. Nat. Biomed. Eng..

[30] Campanella G., Hanna M.G., Geneslaw L., Miraflor A., Werneck Krauss Silva V., Busam K.J., Brogi E., Reuter V.E., Klimstra D.S., Fuchs T.J. (2019). Clinical-grade computational pathology using weakly supervised deep learning on whole slide images. Nat. Med..

[31] Weigelt B., Geyer F.C., Reis-Filho J.S. (2010). Histological types of breast cancer: How special are they?. Mol. Oncol..

[32] Sanchez-Vega F., Mina M., Armenia J., Chatila W.K., Luna A., La K.C., Dimitriadoy S., Liu D.L., Kantheti H.S., Saghafinia S. (2018). Oncogenic Signaling Pathways in The Cancer Genome Atlas. Cell.

[33] He Y., Sun M.M., Zhang G.G., Yang J., Chen K.S., Xu W.W., Li B. (2021). Targeting PI3K/Akt signal transduction for cancer therapy. Sig Transduct Target Ther.

[34] Garmendia I., Redin E., Montuenga L.M., Calvo A. (2022). YES1: A Novel Therapeutic Target and Biomarker in Cancer. Mol. Cancer Ther..

[35] Jiang T., Zhou C., Ren S. (2016). Role of IL-2 in cancer immunotherapy. OncoImmunology.

[36] Carpenter J.E., Clarke C.L. (2014). Biobanking Sustainability—Experiences of the Australian Breast Cancer Tissue Bank (ABCTB). Biopreserv. Biobank..

[37] Hoadley K.A., Yau C., Hinoue T., Wolf D.M., Lazar A.J., Drill E., Shen R., Taylor A.M., Cherniack A.D., Thorsson V. (2018). Cell-of-Origin Patterns Dominate the Molecular Classification of 10,000 Tumors from 33 Types of Cancer. Cell.

[38] Cancer Genome Atlas Network (2012). Comprehensive molecular portraits of human breast tumours. Nature.

[39] Mertins P., Mani D.R., Ruggles K.V., Gillette M.A., Clauser K.R., Wang P., Wang X., Qiao J.W., Cao S., Petralia F. (2016). Proteogenomics connects somatic mutations to signaling in breast cancer. Nature.

[40] Curtis C., Shah S.P., Chin S.F., Turashvili G., Rueda O.M., Dunning M.J., Speed D., Lynch A.G., Samarajiwa S., Yuan Y. (2012). The genomic and transcriptomic architecture of 2,000 breast tumours reveals novel subgroups. Nature.

[41] Pereira B., Chin S.F., Rueda O.M., Vollan H.K.M., Provenzano E., Bardwell H.A., Pugh M., Jones L., Russell R., Sammut S.J. (2016). The somatic mutation profiles of 2,433 breast cancers refines their genomic and transcriptomic landscapes. Nat. Commun..

[42] Cerami E., Gao J., Dogrusoz U., Gross B.E., Sumer S.O., Aksoy B.A., Jacobsen A., Byrne C.J., Heuer M.L., Larsson E. (2012). The cBio cancer genomics portal: an open platform for exploring multidimensional cancer genomics data. Cancer Discov..

[43] Gao J., Aksoy B.A., Dogrusoz U., Dresdner G., Gross B., Sumer S.O., Sun Y., Jacobsen A., Sinha R., Larsson E. (2013). Integrative analysis of complex cancer genomics and clinical profiles using the cBioPortal. Sci. Signal..

[44] Zhang X., Zhou X., Lin M., Sun J. (2018). 2018 IEEE/CVF Conference on Computer Vision and Pattern Recognition.

[45] Clark K., Vendt B., Smith K., Freymann J., Kirby J., Koppel P., Moore S., Phillips S., Maffitt D., Pringle M. (2013). The Cancer Imaging Archive (TCIA): Maintaining and Operating a Public Information Repository. J. Digit. Imaging.

[46] Steeg G.V., Galstyan A. (2015). Maximally Informative Hierarchical Representations of High-Dimensional Data. arXiv.

[47] Kuleshov M.V., Jones M.R., Rouillard A.D., Fernandez N.F., Duan Q., Wang Z., Koplev S., Jenkins S.L., Jagodnik K.M., Lachmann A. (2016). Enrichr: a comprehensive gene set enrichment analysis web server 2016 update. Nucleic Acids Res..

[48] Szklarczyk D., Santos A., von Mering C., Jensen L.J., Bork P., Kuhn M. (2016). STITCH 5: augmenting protein–chemical interaction networks with tissue and affinity data. Nucleic Acids Res..

[49] Keller P., Dawood M., Minhas F. ul A., Chen H., Luo L. (2023). Trustworthy Machine Learning for Healthcare.

[50] Russakovsky O., Deng J., Su H., Krause J., Satheesh S., Ma S., Huang Z., Karpathy A., Khosla A., Bernstein M. (2015). ImageNet Large Scale Visual Recognition Challenge. Int. J. Comput. Vis..

[51] Wang Y., Sun Y., Liu Z., Sarma S.E., Bronstein M.M., Solomon J.M. (2019). Dynamic Graph CNN for Learning on Point Clouds. arXiv.

[52] Dawood M., Branson K., Rajpoot N.M., Ul Amir Afsar Minhas F. (2021). 2021 IEEE/CVF International Conference on Computer Vision Workshops (ICCVW).

[53] Jahanifar M., Shephard A., Zamanitajeddin N., Raza S.E.A., Rajpoot N. (2022). Stain-Robust Mitotic Figure Detection for MIDOG 2022 Challenge. arXiv.

