# Supplementary material for: Cross-linking breast tumor transcriptomic states and tissue histology
Source: Cell Rep Med. 2023 Dec 19;4(12):101313. doi: 10.1016/j.xcrm.2023.101313 (PMC10783602; doi:10.1016/j.xcrm.2023.101313)
Supplement: Document S1. Figures S1–S6 and Tables S1 and S2 [file mmc1.pdf]

Cell Reports Medicine, Volume *4*

Supplemental information

Cross-linking breast tumor transcriptomic states and tissue histology

Muhammad Dawood, Mark Eastwood, Mostafa Jahanifar, Lawrence Young, Asa Ben-Hur, Kim Branson, Louise Jones, Nasir Rajpoot, and Fayyaz ul Amir Afsar Minhas

Cross-linking breast tumor transcriptomic states and tissue histology

Muhammad Dawood, Mark Eastwood, Mostafa Jahanifar, Lawrence Young, Asa Ben-Hur, Kim Branson, Louise Jones, Nasir Rajpoot, Fayyaz ul Amir Afsar Minhas

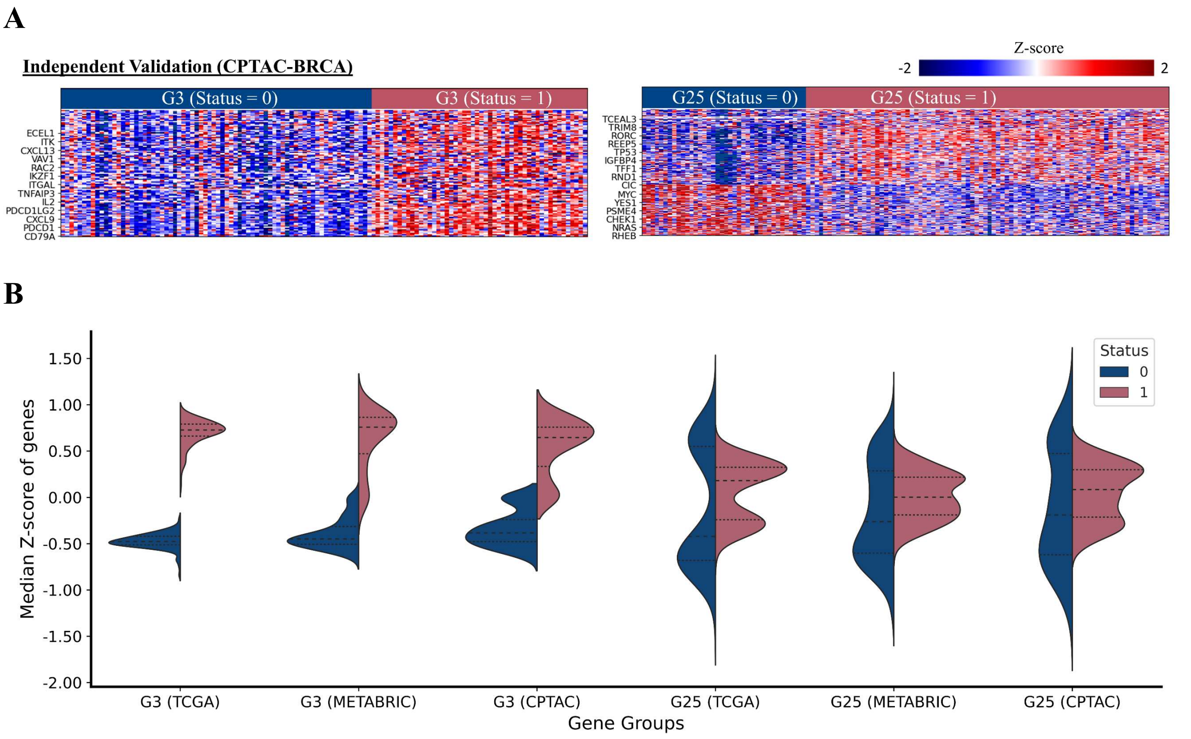

Figure S1: Independent validation of gene group status association with the expression level of genes in the group, related to Figure 2.

A) Gene expression profile and group status of genes (one per row) for all patients (one per column) in Gene Group 3 (G3) is shown.

B) Violin plots showing the median expression of genes in Gene Group 3 (G3) and Gene Group 25 (G25) across three cohorts (TCGA, CPTAC and METABRIC). The dark blue color represents the distribution of median genes expression for patients with status = 0, while red color indicates the distribution for patients with status = 1.

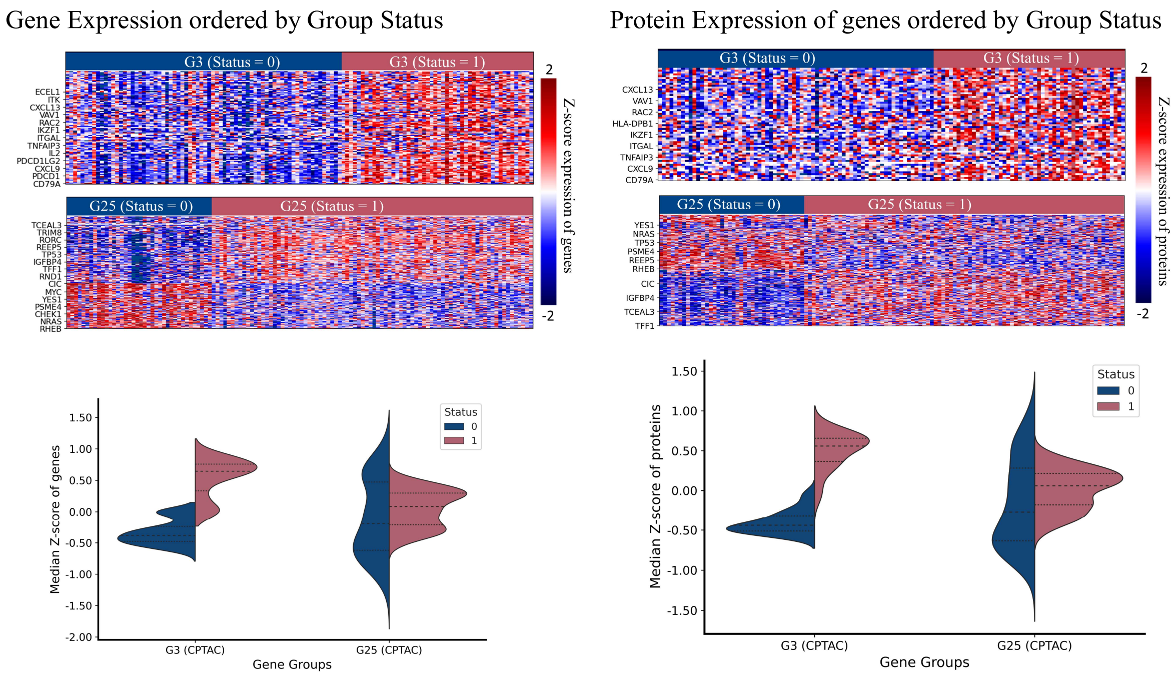

Figure S2: Association of gene groups status with the expression level of proteins, related to Figure 2. Heatmaps showing degree of association between G3 and G25 status of patients in the CPTAC-BRCA cohort with the expression level of genes (left plot) and proteins (right plot) in the group. In the heatmaps genes/proteins are shown along y-axis while patients are arranged by their gene group status along x-axis. The color shows z-score expression of genes/proteins. The violin plot below each heatmap shows the distribution of median expression of genes/proteins for patients with gene groups status being 0 (dark blue color) or 1 (red color).

Independent Validation (CPTAC-BRCA)

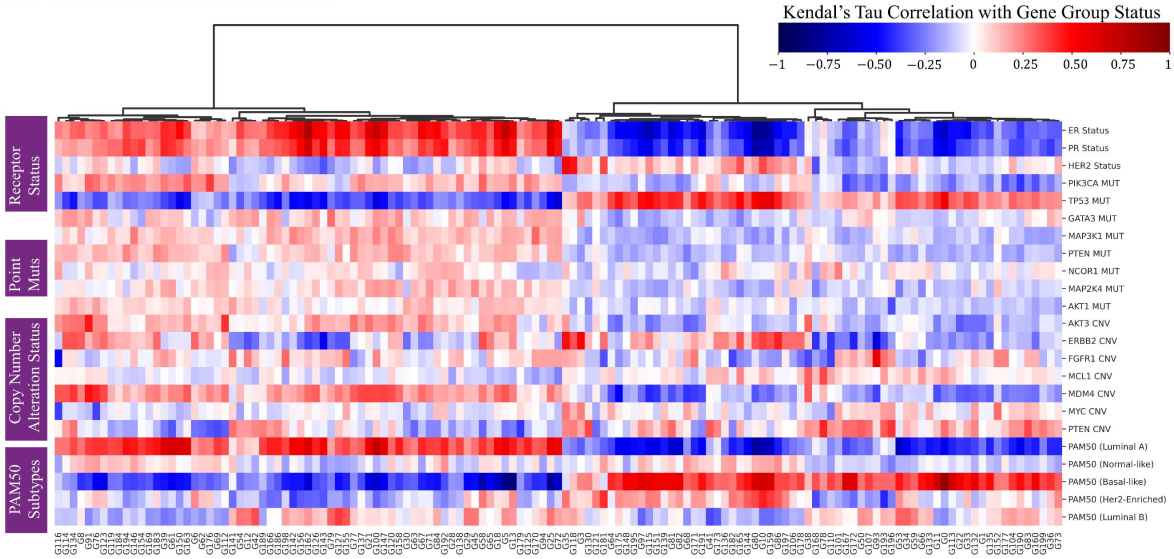

Independent Validation (METABRIC)

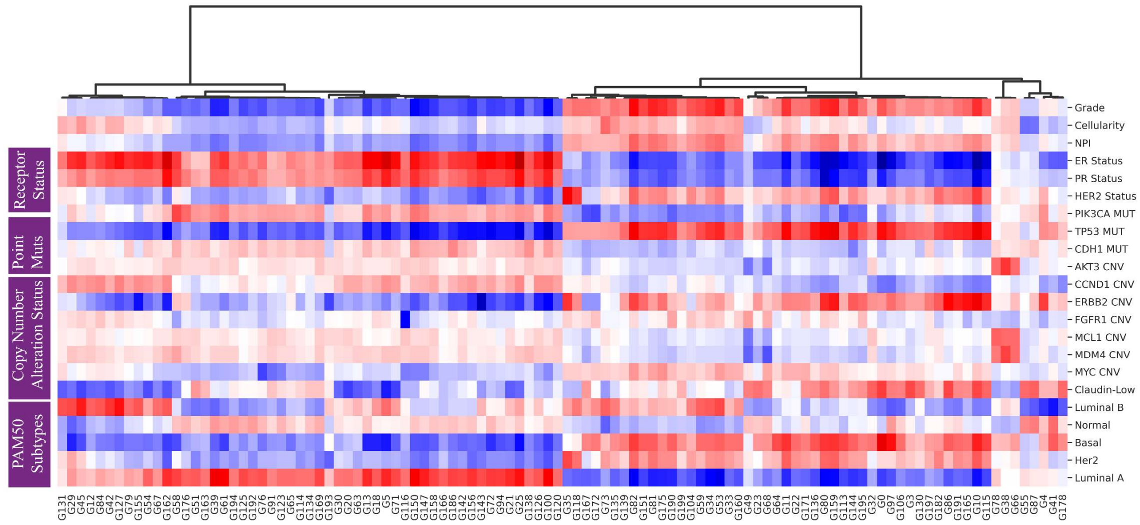

Figure S3: Independent validation of clinical and pathological significance of gene groups on CPTAC-BRCA and METABRIC cohort, related to Figure 3.

The heatmaps validate the consistency of gene groups status association with histological phenotypes, receptor status, genes point mutation status (MUT status) and copy number alteration status (CNA status), and PAM50 molecular subtypes on CPTAC-BRCA (top plot) and METABRIC (bottom plot) cohort. The plots display gene groups along the x-axis and the corresponding clinical variables along the y-axis. Red and blue colors indicate the degree of association between gene groups status and a specific histopathological phenotype or clinical marker. Dark-red color shows strong positive correlation while strong negative correlation is shown using dark-blue color.

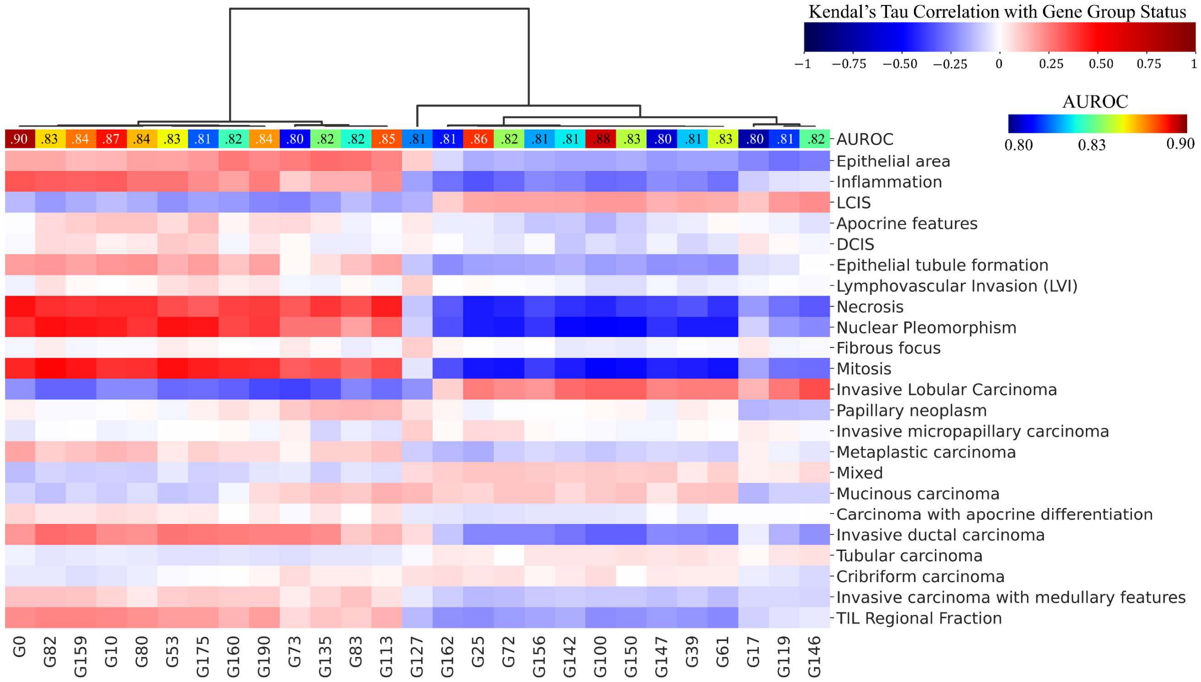

A: Gene groups predicted with high accuracy (AUROC ≥ 0.80).

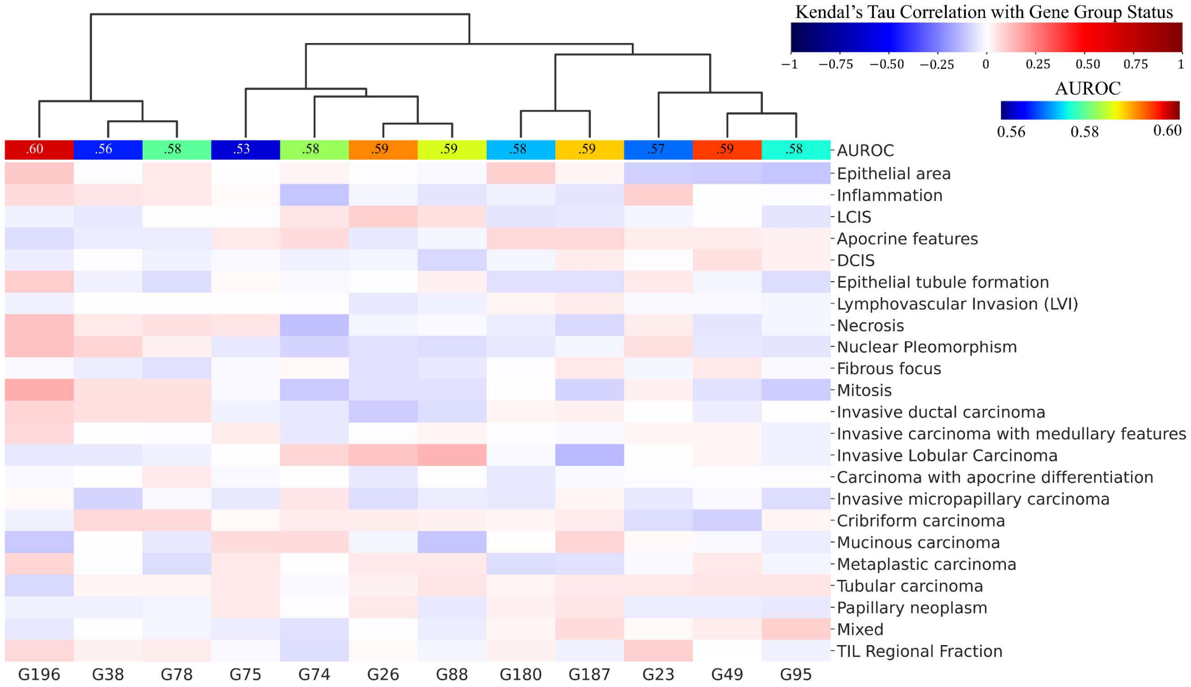

B: Gene groups predicted with poor accuracy (AUROC ≤ 0.60).

Figure S4: Association of binary statuses of best and worst predicted gene groups with pathologist-assigned histological phenotypes, related to Figure 4.

The heatmaps show gene groups predictability from imaging (AUROC) and their association with histological phenotypes (Kendall's Tau correlation). The AUROC values are shown using jet colormap, while the correlation

between gene groups and pathologist-assigned phenotypes are shown using seismic color. The numeric values within the AUROC color band represent the mean AUROC values across test folds for a certain gene group.

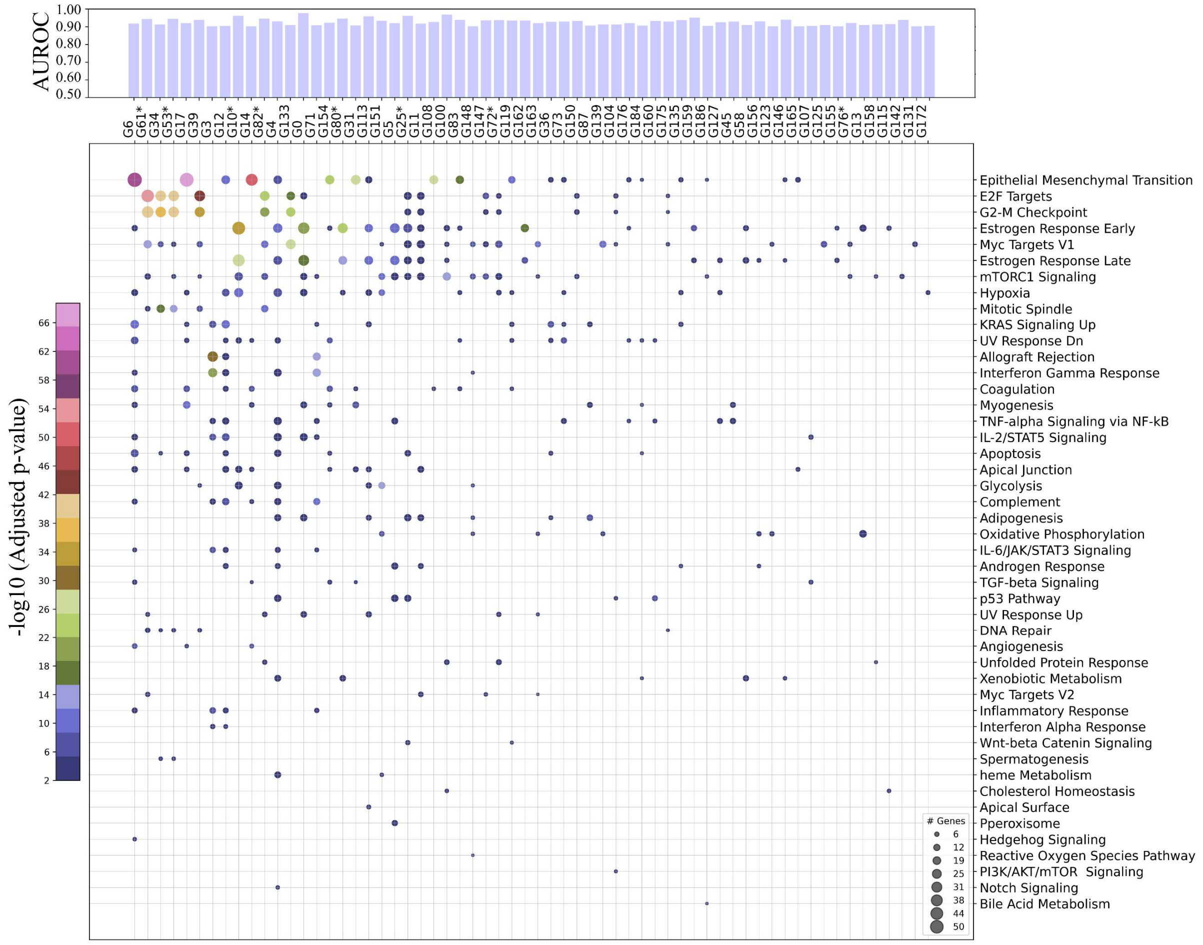

Figure S5: Association of best-predicted gene groups (AUROC ≥ 0.75) with cancer Hallmark processes and disease-specific survival, related to Figure 8.
An example 2D scatter plot showing gene groups (one per column) association with hallmark processes (one per row). The size of the scatter dot indicates the number of genes in a gene group that have shown enrichment (FDR adjusted p-value < 0.01) for a certain cancer hallmark process, while the dot color indicates the p-value. The top bar plot shows the prediction accuracy (AUROC) at which the status of these gene groups are predicted from histology images. Gene groups that show significant association with disease specific survival are annotated with a * next to the gene group name.

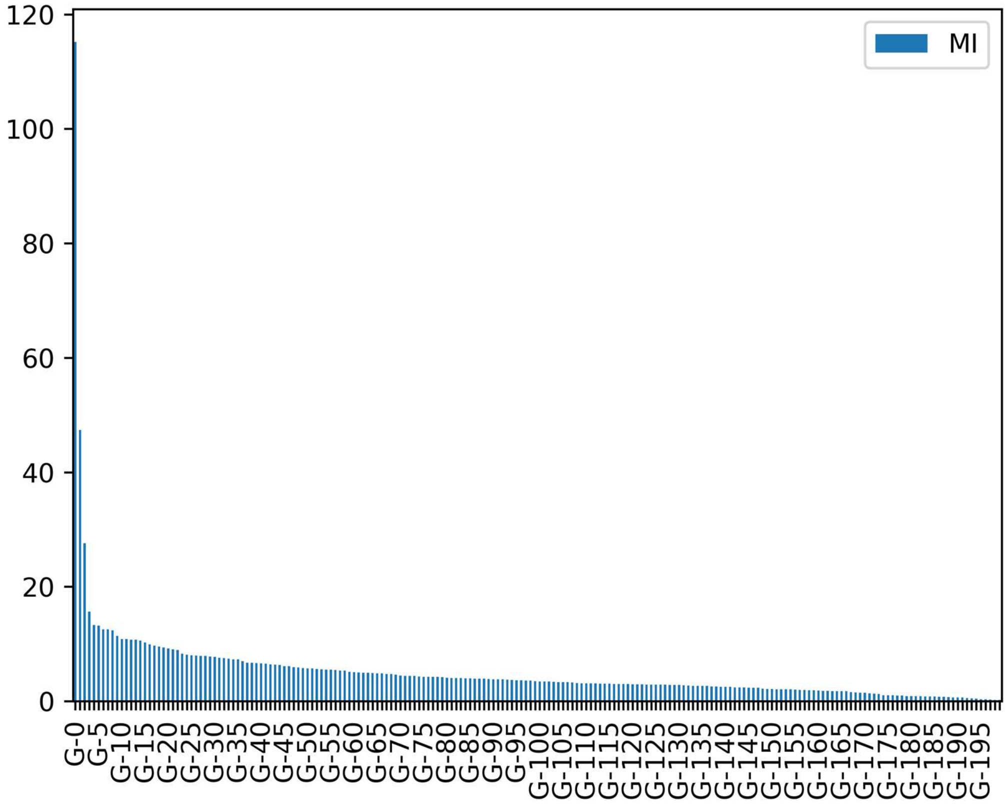

Figure S6: Plot showing the proportion of total correlation explained by each latent factor, related to **STAR Methods** (Data Driven Discovery of Gene Groups with CorEx).

Table S1: Analysis of potential batch effects and other confounders, related to Figure 2.
The table summarizes the degree of predictability of tissue source site using gene groups status using AUROC as performance metric. The first column lists tissue source sites, the (+, -) column lists the (samples belonging to a certain site, rest of samples), followed by the mean AUROC of SVM and XGBoost classifier across five non-redundant validation splits.

**Source Site**	**(+,-)**	**SVM (AUROC ± STD)**	**XGBoost (AUROC ± STD)**
A1	(347, 735)	0.624 ± 0.055	0.657 ± 0.054
A2	(234,848)	0.536 ± 0.027	0.547 ± 0.028
BH	(150,932)	0.533 ± 0.058	0.579 ± 0.061
A8	(99,983)	0.578 ± 0.065	0.594 ± 0.134
E2	(89,993)	0.504 ± 0.063	0.474 ± 0.078
D8	(78,1004)	0.695 ± 0.059	0.647 ± 0.052
AR	(69,1013)	0.605 ± 0.057	0.609 ± 0.111
E9	(63,1019)	0.531 ± 0.092	0.495 ± 0.045
B6	(59,1023)	0.562 ± 0.061	0.643 ± 0.042
A7	(58,1024)	0.512 ± 0.085	0.511 ± 0.151

**Table S2: Thumbnails of patient WSIs whose gene expression state are best or poorly predicted from histology images, related to Figure 4.** The first column shows thumbnails of patients' WSIs whose predicted and ground truth gene expression state show good alignment in terms of cosine similarity (S_c), while the second column shows thumbnails of patients' WSIs whose predicted and ground truth expression state show poor alignment.			
**Best Predicted**		**Worst Predicted**	
Thumbnails	S_c	Thumbnails	S_c
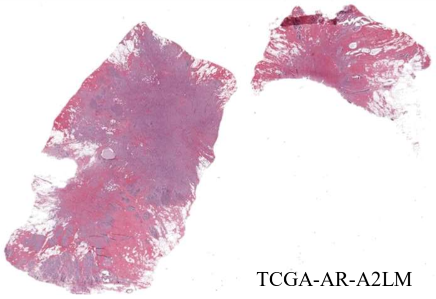 TCGA-AR-A2LM	0.84	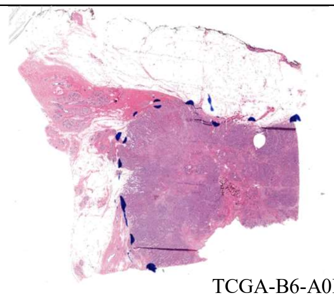 TCGA-B6-A0I2	-0.45
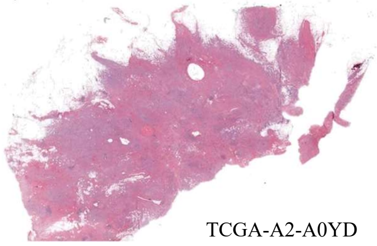 TCGA-A2-A0YD	0.82	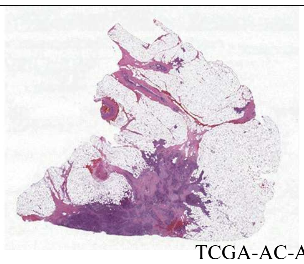 TCGA-AC-A7VB	-0.30
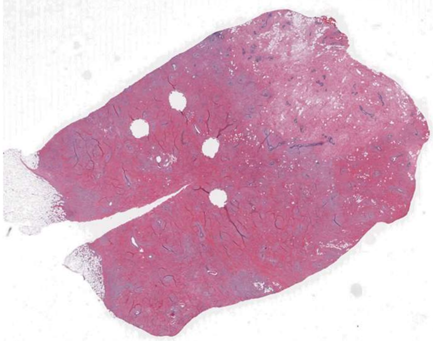 TCGA-AR-A1AL	0.79	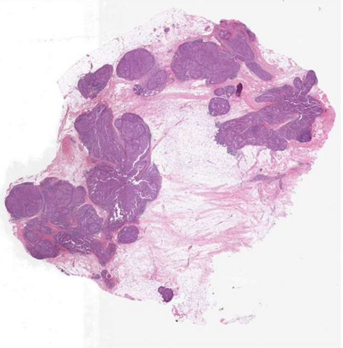 TCGA-AC-A3YJ	-0.099
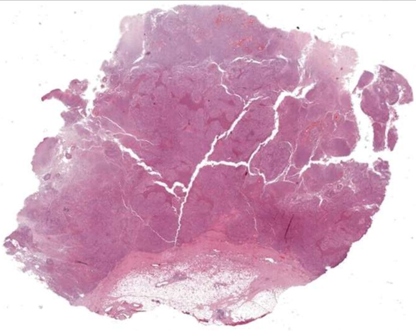 TCGA-D8-A1XK	0.78	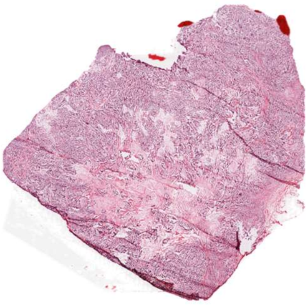 TCGA-E9-A1N8	0.18
