# Supplementary material for: Cross-linking breast tumor transcriptomic states and tissue histology
Source: Cell Rep Med. 2023 Dec 19;4(12):101313. doi: 10.1016/j.xcrm.2023.101313 (PMC10783602; doi:10.1016/j.xcrm.2023.101313)
Supplement: Data S1. Gene Groups status of patients in TCGA-BRCA, CPTAC-BRCA and METABRIC cohorts, related to Figure 2A data-driven discovery of gene groups [file mmc2.pdf]

Cross-linking breast tumor transcriptomic states and tissue histology

Muhammad Dawood, Mark Eastwood, Mostafa Jahanifar, Lawrence Young, Asa Ben-Hur, Kim Branson, Louise Jones, Nasir Rajpoot, Fayyaz ul Amir Afsar Minhas

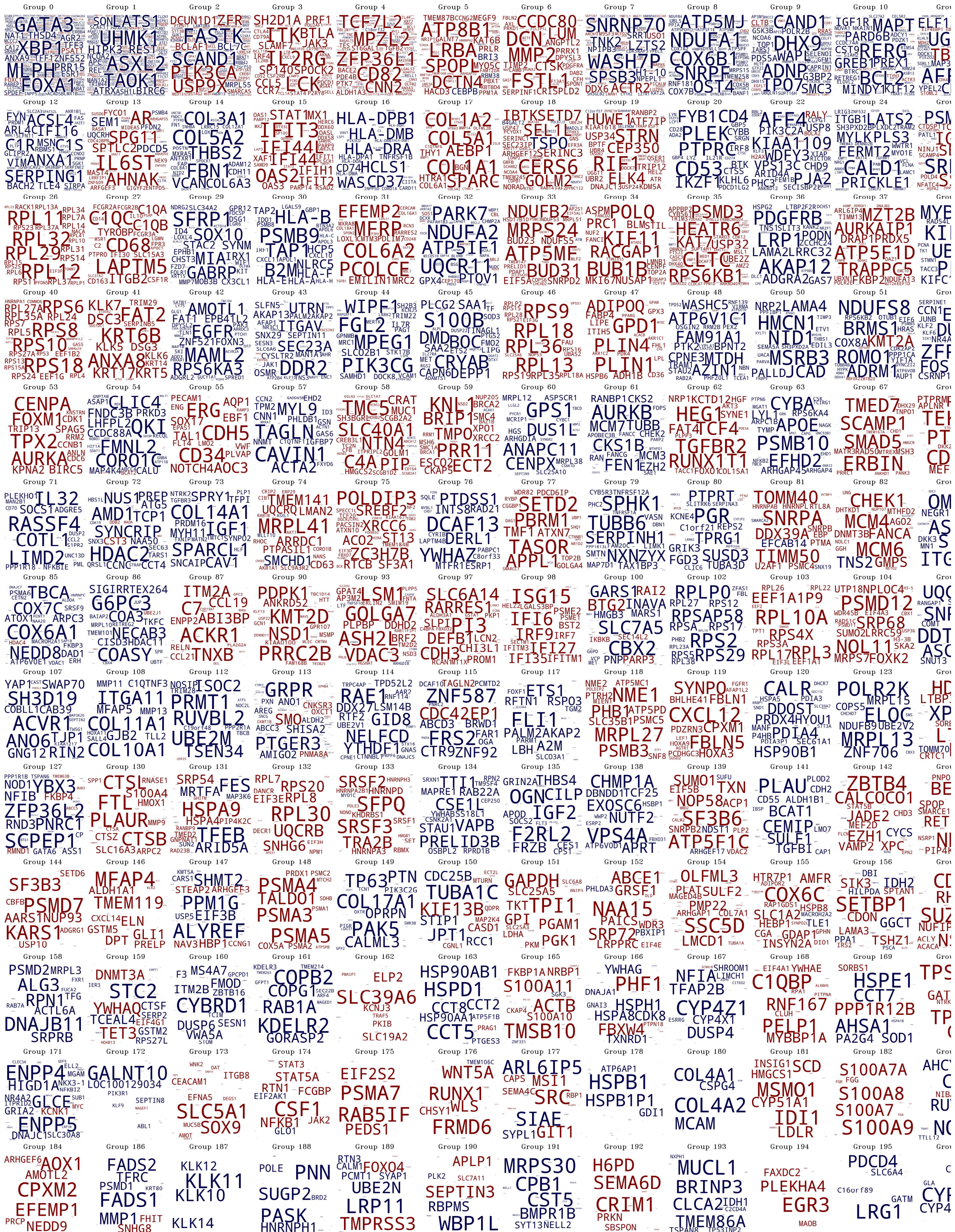

Word clouds demonstrating the gene composition of different gene groups. The color of the gene indicates whether its median expression across patients is high (red) or low (blue) when gene group status = 1. The font size of gene within a group is proportional to the amount of information that the gene status provides about a particular gene.

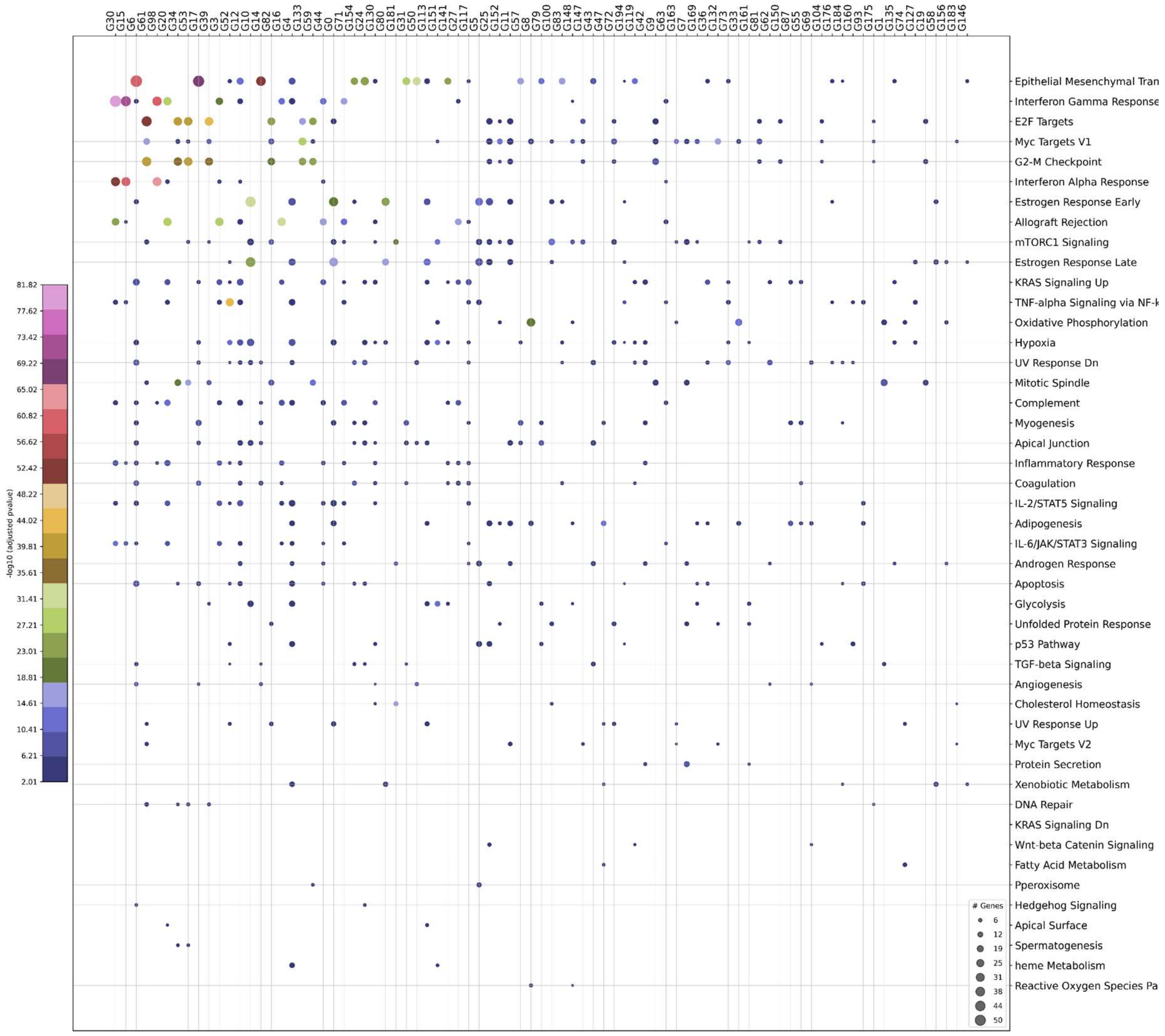

Enrichment of gene groups for cancer Hallmark processes is illustrated as 2D scatter plot with the gene group displayed along x-axis and the corresponding enriched biological pathways on y-axis. The size of the dot represents the number of genes from a specific gene group that has shown enrichment for a particular hallmark process while its color represents the statistical significance of association in terms of FDR-corrected p-value.

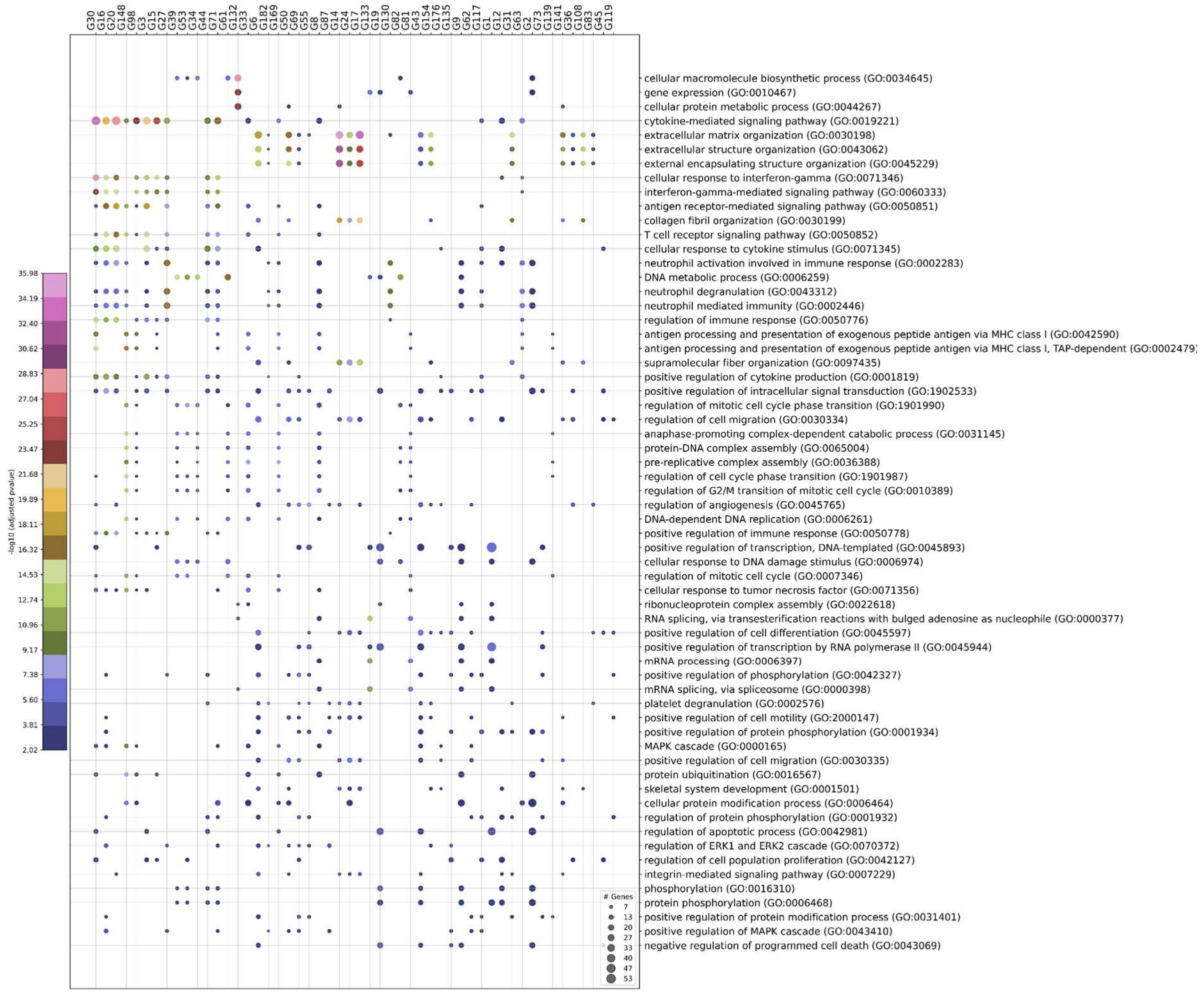

Enrichment of gene groups for GO (Gene ontology) biological processes is shown as 2D scatter plot with the gene groups displayed along x-axis and the corresponding enriched biological processes on y-axis. The size of scatter dot represents the number of genes from a specific gene group that has shown enrichment for a particular biological process while its color represents the statistical significance of the association in terms of FDR-corrected p-value.

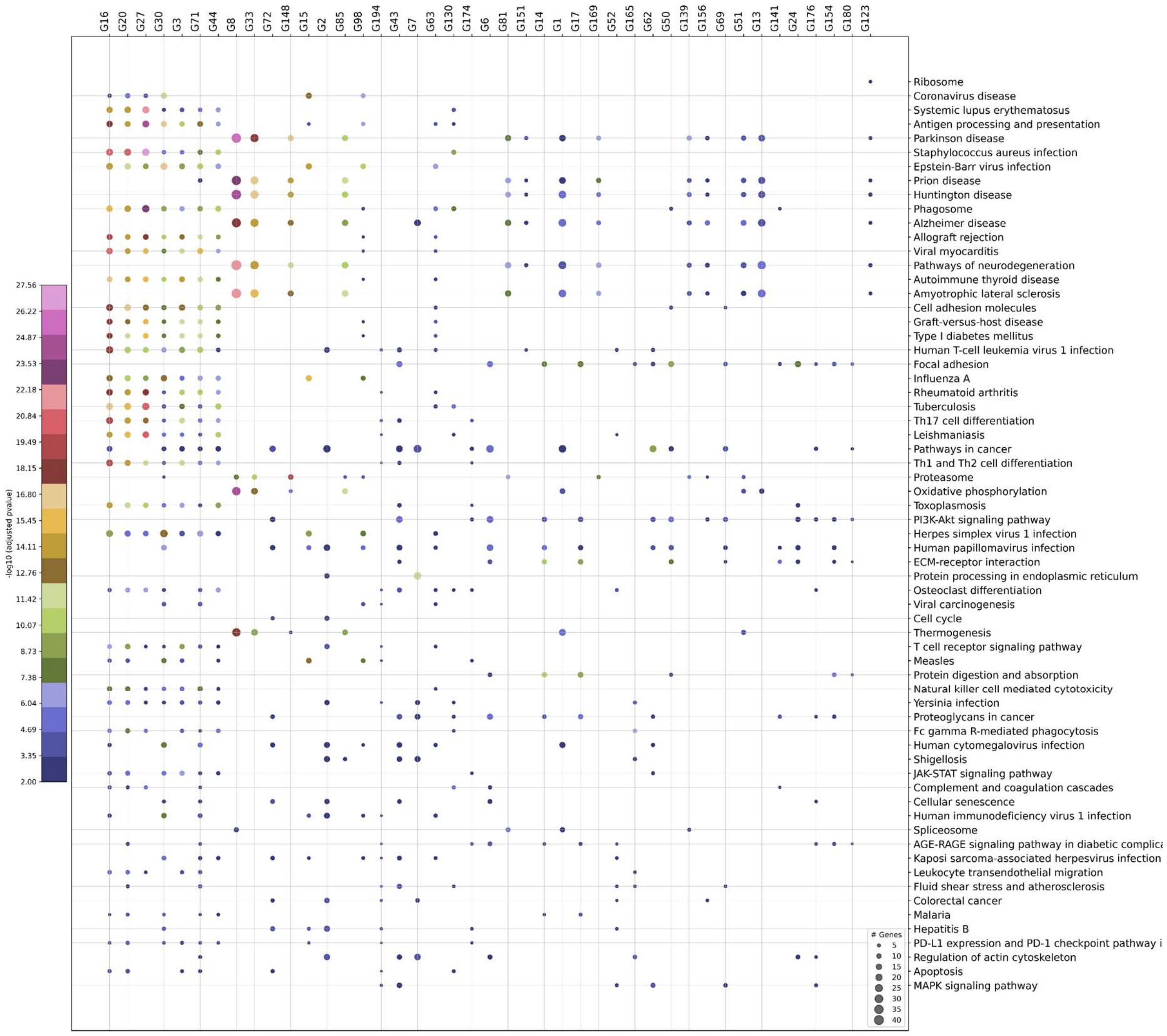

Enrichment of gene groups for KEGG Pathways is presented as 2D scatter plot with the gene group displayed along x-axis and the corresponding enriched biological pathways on y-axis. The size of the dot represents the number of genes from a specific gene group that has shown enrichment for a particular biological pathway while its color represents the statistical significance of association in terms of FDR-corrected p-value.

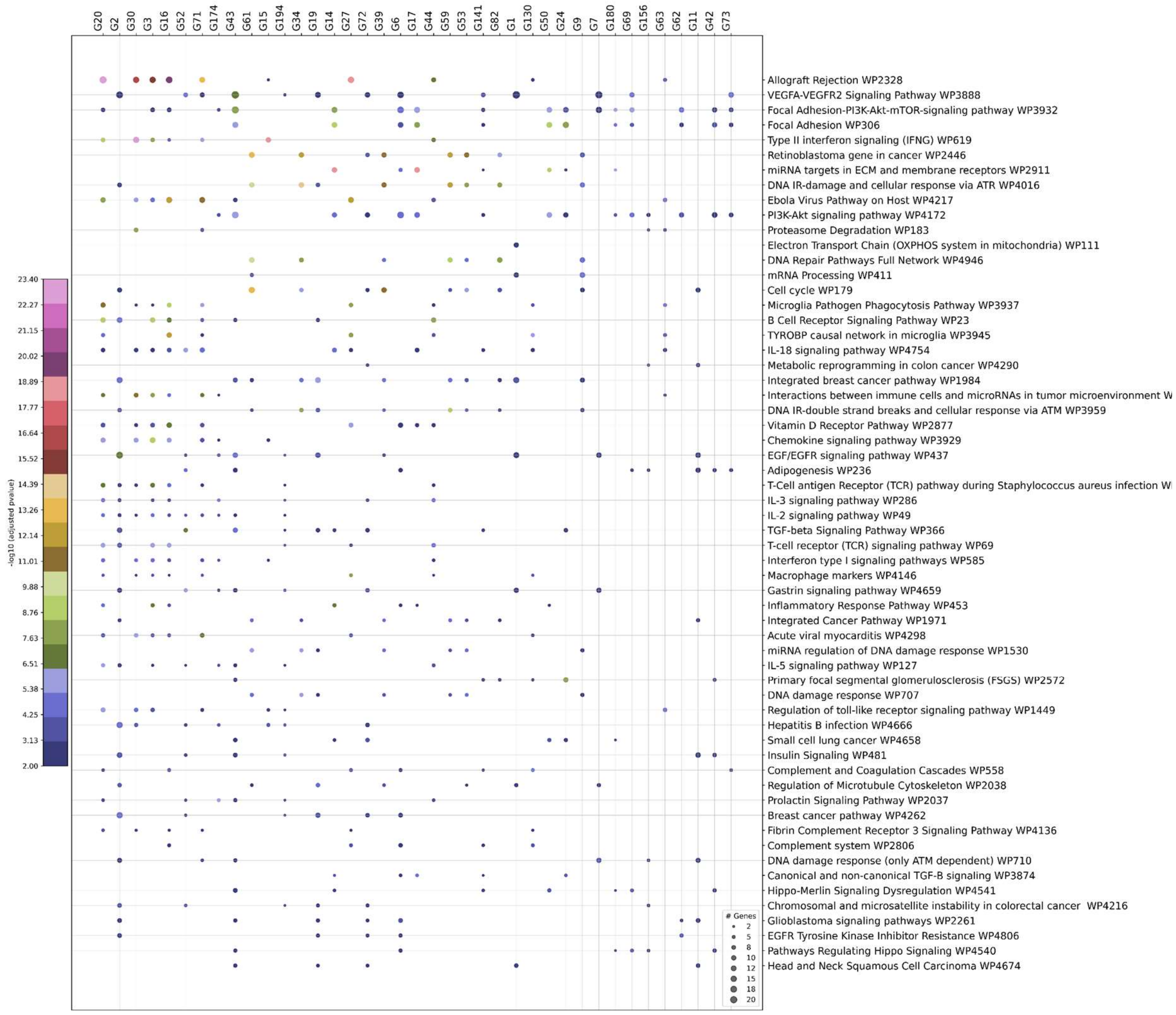

Enrichment of gene groups for cancer WikiPathways is illustrated as 2D scatter plot with the gene group displayed along x-axis and the corresponding enriched pathways on y-axis. The size of the dot represents the number of genes from a specific gene group that has shown enrichment for a particular pathway while its color represents the statistical significance of association in terms of FDR-corrected p-value.
